# Identification of a Sjögren's syndrome susceptibility locus at *OAS1* that influences isoform switching, protein expression, and responsiveness to type I interferons

**DOI:** 10.1371/journal.pgen.1006820

**Published:** 2017-06-22

**Authors:** He Li, Tove Ragna Reksten, John A. Ice, Jennifer A. Kelly, Indra Adrianto, Astrid Rasmussen, Shaofeng Wang, Bo He, Kiely M. Grundahl, Stuart B. Glenn, Corinne Miceli-Richard, Simon Bowman, Sue Lester, Per Eriksson, Maija-Leena Eloranta, Johan G. Brun, Lasse G. Gøransson, Erna Harboe, Joel M. Guthridge, Kenneth M. Kaufman, Marika Kvarnström, Deborah S. Cunninghame Graham, Ketan Patel, Adam J. Adler, A. Darise Farris, Michael T. Brennan, James Chodosh, Rajaram Gopalakrishnan, Michael H. Weisman, Swamy Venuturupalli, Daniel J. Wallace, Kimberly S. Hefner, Glen D. Houston, Andrew J. W. Huang, Pamela J. Hughes, David M. Lewis, Lida Radfar, Evan S. Vista, Contessa E. Edgar, Michael D. Rohrer, Donald U. Stone, Timothy J. Vyse, John B. Harley, Patrick M. Gaffney, Judith A. James, Sean Turner, Ilias Alevizos, Juan-Manuel Anaya, Nelson L. Rhodus, Barbara M. Segal, Courtney G. Montgomery, R. Hal Scofield, Susan Kovats, Xavier Mariette, Lars Rönnblom, Torsten Witte, Maureen Rischmueller, Marie Wahren-Herlenius, Roald Omdal, Roland Jonsson, Wan-Fai Ng, for UK Primary Sjögren's Syndrome Registry, Gunnel Nordmark, Christopher J. Lessard, Kathy L. Sivils

**Affiliations:** 1 Arthritis and Clinical Immunology Research Program, Oklahoma Medical Research Foundation, Oklahoma City, Oklahoma, United States of America; 2 Department of Pathology, University of Oklahoma Health Sciences Center, Oklahoma City, Oklahoma, United States of America; 3 Broegelmann Research Laboratory, Department of Clinical Science, University of Bergen, Bergen, Norway; 4 Université Paris-Sud, AP-HP, Hôpitaux Universitaires Paris-Sud, INSERM U1012, Le Kremlin Bicêtre, France; 5 Rheumatology Department, University Hospital Birmingham, Birmingham, United Kingdom; 6 The Queen Elizabeth Hospital, Adelaide, South Australia, Australia; 7 Department of Rheumatology, Clinical and Experimental Medicine, Linköping University, Linköping, Sweden; 8 Department of Medical Sciences, Rheumatology, SciLIfeLab, Uppsala University, Uppsala, Sweden; 9 Department of Clinical Science, University of Bergen, Bergen, Norway; 10 Department of Rheumatology, Haukeland University Hospital, Bergen, Norway; 11 Clinical Immunology Unit, Department of Internal Medicine, Stavanger University Hospital, Stavanger, Norway; 12 Division of Rheumatology, Cincinnati Children’s Hospital Medical Center, Cincinnati, Ohio, United States of America; 13 US Department of Veterans Affairs Medical Center, Cincinnati, Ohio, United States of America; 14 Department of Medicine, Karolinska Institutet, Stockholm, Sweden; 15 Department of Medical and Molecular Genetics, King's College London, London, United Kingdom; 16 Division of Oral and Maxillofacial Surgery, Department of Developmental and Surgical Science, University of Minnesota School of Dentistry, Minneapolis, Minnesota, United States of America; 17 Department of Oral and Maxillofacial Surgery, North Memorial Medical Center, Robbinsdale, Minnesota, United States of America; 18 Department of Oral Medicine, Carolinas Medical Center, Charlotte, North Carolina, United States of America; 19 Massachusetts Eye and Ear Infirmary, Department of Ophthalmology, Harvard Medical School, Boston, Massachusetts, United States of America; 20 Division of Oral Pathology, Department of Diagnostic and Biological Sciences, University of Minnesota School of Dentistry, Minneapolis, Minnesota, United States of America; 21 Division of Rheumatology, Cedars-Sinai Medical Center, Los Angeles, California, United States of America; 22 Hefner Eye Care and Optical Center, Oklahoma City, Oklahoma, United States of America; 23 Department of Oral and Maxillofacial Pathology, University of Oklahoma College of Dentistry, Oklahoma City, Oklahoma, United States of America; 24 Heartland Pathology Consultants, Edmond, Oklahoma, United States of America; 25 Department of Ophthalmology and Visual Sciences, Washington University, St. Louis, Missouri, United States of America; 26 Oral Diagnosis and Radiology Department, University of Oklahoma College of Dentistry, Oklahoma City, Oklahoma, United States of America; 27 University of Santo Tomas Hospital, Manila, The Philippines; 28 The Biology Department, Oklahoma Baptist University, Oklahoma City, Oklahoma, United States of America; 29 Hard Tissue Research Laboratory, University of Minnesota School of Dentistry, Minneapolis, Minnesota, United States of America; 30 Department of Ophthalmology, Johns Hopkins University, Baltimore, Maryland, United States of America; 31 Department of Medicine, University of Oklahoma Health Sciences Center, Oklahoma City, Oklahoma, United States of America; 32 National Institute of Dental and Craniofacial Research, NIH, Bethesda, Maryland, United States of America; 33 Center for Autoimmune Diseases Research, Universidad del Rosario, Bogotá, Colombia; 34 Department of Oral Surgery, University of Minnesota School of Dentistry, Minneapolis, Minnesota, United States of America; 35 Division of Rheumatology, University of Minnesota Medical School, Minneapolis, Minnesota, United States of America; 36 US Department of Veterans Affairs Medical Center, Oklahoma City, Oklahoma, United States of America; 37 Clinic for Immunology and Rheumatology, Hannover Medical School, Hannover, Germany; 38 The University of Adelaide, Adelaide, South Australia, Australia; 39 Institute of Cellular Medicine & NIHR Newcastle Biomedical Research Centre, Newcastle University, Newcastle upon Tyne, United Kingdom; Gronigen University, NETHERLANDS

## Abstract

Sjögren’s syndrome (SS) is a common, autoimmune exocrinopathy distinguished by keratoconjunctivitis sicca and xerostomia. Patients frequently develop serious complications including lymphoma, pulmonary dysfunction, neuropathy, vasculitis, and debilitating fatigue. Dysregulation of type I interferon (IFN) pathway is a prominent feature of SS and is correlated with increased autoantibody titers and disease severity. To identify genetic determinants of IFN pathway dysregulation in SS, we performed *cis*-expression quantitative trait locus (eQTL) analyses focusing on differentially expressed type I IFN-inducible transcripts identified through a transcriptome profiling study. Multiple *cis*-eQTLs were associated with transcript levels of 2'-5'-oligoadenylate synthetase 1 (*OAS1*) peaking at rs10774671 (*P*_*eQTL*_ = 6.05 × 10^−14^). Association of rs10774671 with SS susceptibility was identified and confirmed through meta-analysis of two independent cohorts (*P*_*meta*_ = 2.59 × 10^−9^; odds ratio = 0.75; 95% confidence interval = 0.66–0.86). The risk allele of rs10774671 shifts splicing of *OAS1* from production of the p46 isoform to multiple alternative transcripts, including p42, p48, and p44. We found that the isoforms were differentially expressed within each genotype in controls and patients with and without autoantibodies. Furthermore, our results showed that the three alternatively spliced isoforms lacked translational response to type I IFN stimulation. The p48 and p44 isoforms also had impaired protein expression governed by the 3' end of the transcripts. The SS risk allele of rs10774671 has been shown by others to be associated with reduced OAS1 enzymatic activity and ability to clear viral infections, as well as reduced responsiveness to IFN treatment. Our results establish *OAS1* as a risk locus for SS and support a potential role for defective viral clearance due to altered IFN response as a genetic pathophysiological basis of this complex autoimmune disease.

## Author summary

Sjögren’s syndrome (SS) is a common autoimmune condition where immune cells infiltrate moisture-producing glands, leading to dryness typically in the eyes and mouth. SS patients also manifest debilitating fatigue as well as other diseases in liver, lung, kidney, and skin. The cause of this complex disease is still not fully understood; however, an environmental trigger, such as viral infections, in individuals with genetic risk factor(s) is thought to contribute to the development of SS. Type 1 interferons (IFNs) are one of the first defenders after viral infection and induce the expression of various virus-responding genes. Perpetual elevation of type 1 IFN signaling has been observed in SS patients. Here, we first replicated previously identified RNA transcripts contributing to the abnormal type 1 IFN signaling in SS patients. We then identified a disease-associated genetic variant in an IFN-inducible gene, *OAS1*. This variant governs splicing of *OAS1*, altering the transcript into multiple isoforms that lack protein expression and responsiveness to IFNs. The results of this study may provide insight into the genetic basis of SS, as well as other autoimmune disease with similar dysregulation in the type 1 IFN system.

## Introduction

Sjögren’s syndrome (SS) is a common systemic autoimmune disease with a prevalence rate (~0.7% of European Americans) second only to rheumatoid arthritis (RA) [1]. SS is distinguished by immune cell infiltration, functional destruction, and irreversible dysfunction of exocrine glands, most notably salivary and lacrimal glands [2]. Secondary manifestations of exocrine gland dysfunction may include severe dental decay and corneal scarring. Approximately one-third of patients experience extra-glandular manifestations of disease, such as debilitating fatigue, a 16-fold increased risk of developing lymphoma, neuropathies, Raynaud’s phenomenon, arthralgia, and dermatologic symptoms [2–6].

Both glandular dysfunction and extra-glandular manifestations are associated with autoantibodies, a hallmark of autoimmunity [7–9]. Approximately 70% and 40% of SS patients exhibit autoantibodies targeting ribonucleoproteins, Ro/SSA (Ro52 and Ro60) and La/SSB, respectively [10]. These autoantibodies have the capacity to bind necrotic and apoptotic material, thus creating RNA-immune complexes that can activate cells of the immune system and aggravate autoinflammation [11]. Such RNA-containing immune complexes are taken up by the Fc gamma receptor IIa on plasmacytoid dendritic cells (pDCs) [12], which activates intracellular Toll-like receptors 7 and 9 and stimulates type I interferon (IFN) responsive loci [13].

The etiology of SS is still largely unknown, though it involves a complex interplay between both genetic and environmental factors [14–16]. Viral infections, such as Epstein-Barr virus (EBV) and cytomegalovirus [17–19], may initiate prolonged inflammation in glandular lesions and formation of germinal center-like structures commonly linked to autoantibody production in SS [14, 19]. Autoantibodies can be detected up to 18–20 years prior to diagnosis in 81% of SS patients [20]. Indeed, cross-reactivity between antibodies against EBV and the Ro60 antigen has previously been reported [21], and possible subclinical reactivation of the virus has been associated with active joint involvement in SS [22]. Recently, the virus-like genomic repeat element L1 was identified as an endogenous trigger of the IFN pathway, and its expression correlates with type I IFN expression and L1 DNA demethylation [23].

Type I IFNs are key antiviral immune mediators of innate immune responses in infected cells, while at the same time enhancing antigen presentation and inducing production of pro-inflammatory cytokines and chemokines [24], thus initiating adaptive immunity [25]. Overexpression of type I IFN-inducible genes, known as “the IFN signature”, is a common feature of many autoimmune diseases [26, 27], including RA patients with poor clinical outcome [28–30] and systemic lupus erythematosus [31, 32], where the predominant IFN producing cells, pDCs, are reduced in number in the blood but are abundant in skin and lymph nodes [33]. In SS, the IFN signature is observed in both peripheral blood and salivary glands [12, 34–37], and associates with systemic manifestations, greater disease severity, and autoantibody titers [8, 9]. It has been proposed that viral infections contribute to perpetual activation of type I IFN signaling and the resulting dysregulation of innate immunity, ultimately resulting in activation of the adaptive immune response and autoantibody production in SS and other autoimmune diseases [34, 38].

Genome-wide association [GWA] studies in autoimmune diseases have identified multiple genetic risk variants involved in type I IFN signaling pathways [39–41], including associations of *IRF5* and *STAT4* with SS susceptibility [15, 42, 43]. Suggestive associations of *FCGR2A*, *PRDM1* (PR domain containing 1, regulated by *IRF5*) [44], and *IRF8* with SS have also been reported [15], along with two genes within the NF-κB pathway (*TNIP1* and *TNFAIP3*), which regulates early phase type I IFN production during viral infection [45]. Despite the evidence indicating an important role of the type I IFN pathway in SS, no direct functional mechanisms for SS-associated variants contributing to the substantial upregulation of IFN signature transcripts have been described.

The vast majority of disease associated single-nucleotide polymorphisms (SNPs) identified in GWA studies are non-coding [46] and are not likely to impact protein function directly, thus requiring a combination of genetic studies and gene expression analyses to point towards mechanisms that link genetics with functional effects [47]. Specifically, the extensive linkage disequilibrium (LD) observed between associated polymorphisms renders it hard to identify causal variant(s) of disease. Systemic evaluation of genome-wide functional elements by the Encyclopedia of DNA Elements (ENCODE) project reveals that 80% of the human genome has at least one biochemical function, and many of the genetic variants are within *cis*- or *trans*- regulatory sites that impact gene expression [48]. Furthermore, genome-wide *cis*-expression quantitative trait locus (eQTL) mapping studies in different tissues have identified more than 3,000 genes associated with nearby genetic variants [49, 50].

**Fig 1:**
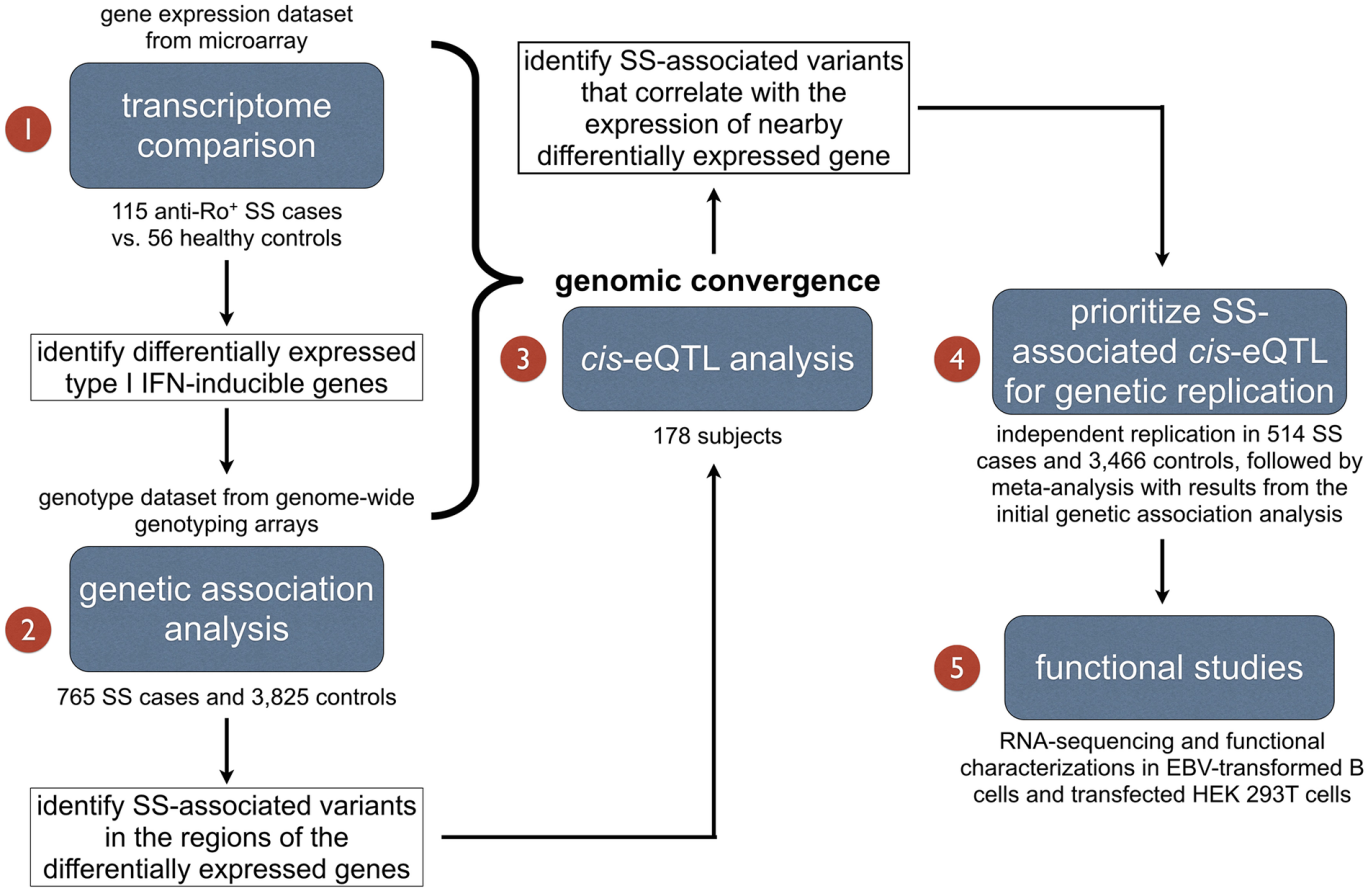
Study design. To evaluate genetic factors involved in the dysregulation of type I IFN signaling in SS, we first compared transcriptional profiles between anti-Ro/SSA positive SS cases and controls to identify genes that make up the IFN signature in SS. We then performed genetic association analysis for variants in the regions of the differentially expressed genes. By integrating transcriptome data with genotype data, *cis*-eQTL analysis was performed for SS-associated SNPs to evaluate their role in gene dysregulation. This genomic convergence approach resulted in increased power to identify and prioritize disease susceptibility genes for further genetic replication and functional studies.

Through combining GWA and gene expression data from SS patients, we sought to identify and characterize SS-associated variants that influence the expression of genes within the IFN signature by utilizing a genomic convergence approach (Fig 1). Through *cis*-eQTL analyses we identified an association of a SNP rs10774671, located within the *OAS1* gene locus, with SS. Functional studies were performed to assess biological consequence of the *OAS1* variants.

## Results

### IFN signature genes identified by transcriptome profiling analysis

**Fig 2:**
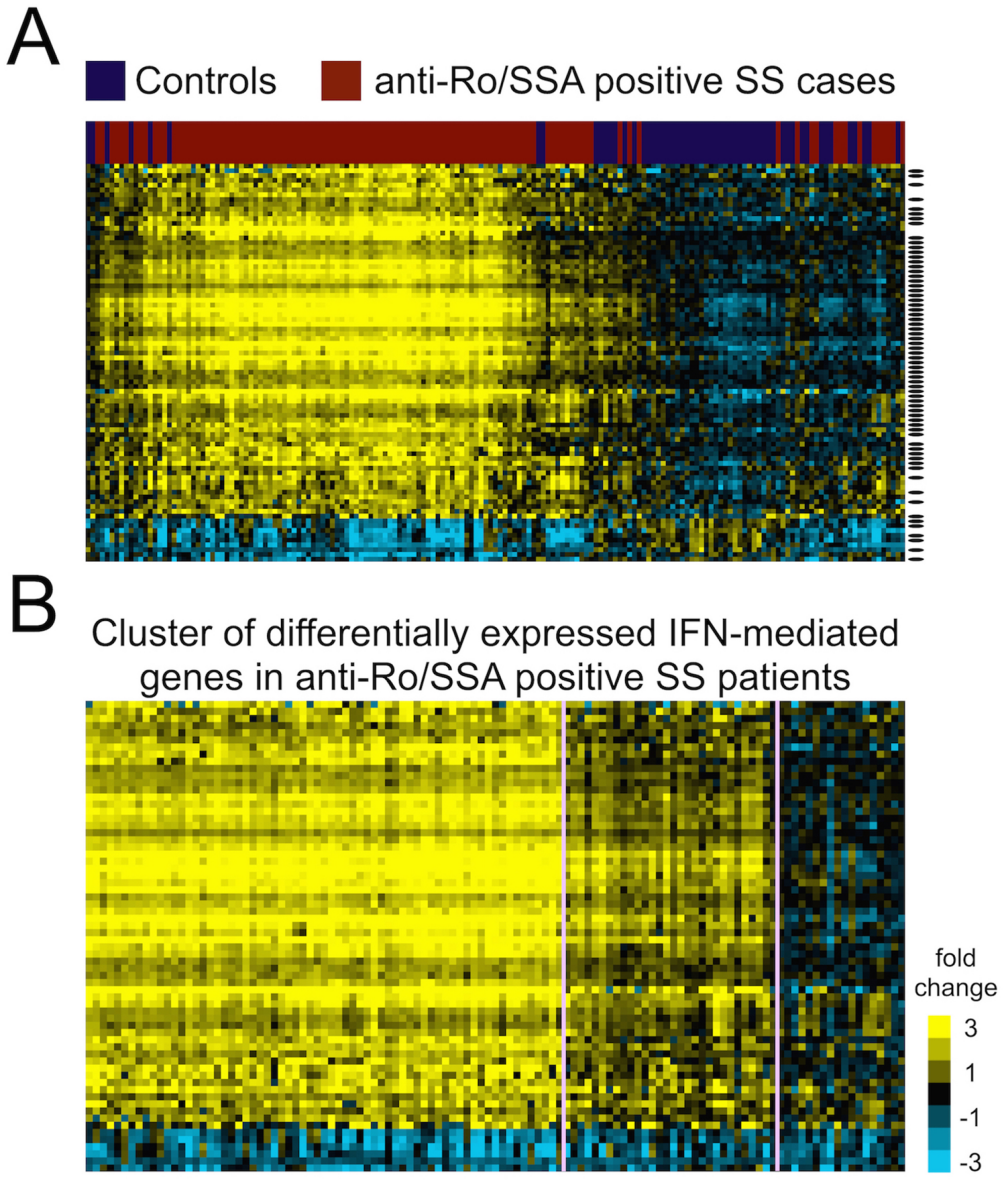
Differentially expressed transcripts between 115 anti-Ro/SSA positive SS cases and 56 controls identified through transcriptome profiling. (A) We identified 73 genes (represented by 83 probes on the heatmap) differentially expressed between anti-Ro/SSA positive SS cases and healthy controls (absolute FC >2 and *q*<0.05). Among the differentially expressed genes, 57 were type I IFN-regulated genes (black bar on right) and formed an IFN signature where most genes were overexpressed in SS patients (yellow indicates overexpressed genes compared to controls). (B) The 57 differentially expressed type I IFN-regulated genes were re-clustered in anti-Ro/SSA positive SS cases using *k*-means (*k* = 3) algorithm and heterogeneity of the IFN signature levels in anti-Ro/SSA positive SS cases was observed.

To select candidate genes in the IFN signature, we first evaluated dysregulated transcripts in SS through a microarray-based gene expression profiling study. Whole blood transcriptome profiles from 115 anti-Ro/SSA positive SS cases and 56 healthy controls of European ancestry were compared, as the IFN signature is enriched in SS patients seropositive for anti-Ro/SSA [34]. After quality control (QC) and normalization, 13,893 probes (in 10,966 genes) remained, for which Welch’s *t*-tests, false discovery rate (FDR)-adjusted *P* values (*q* values), and fold changes (FC; the difference of the mean between log_2_-transformed values from cases and controls) were calculated (see Methods for details). Differentially expressed genes were selected by *q* < 0.05 and FC > 2 or < -2. We found 73 differentially expressed genes in our dataset, among which 57 genes are regulated by type I IFNs (S1 Table). The majority of dysregulated genes (66 out of 73) were overexpressed in SS patients and formed the IFN signature in cases after unsupervised hierarchical clustering (Fig 2A). Of note, the IFN signature was observed in most but not all anti-Ro/SSA positive SS cases, in accordance with our previous work [34], and the intensity of this feature was heterogeneous among patients (Fig 2B). These results suggest that the expression of IFN signature genes might be influenced by genetic variants, which could be identified through *cis*-eQTL analysis.

### Identification of SNP-SS association with rs10774671 in the locus *OAS1*

We hypothesized that variants near the differentially expressed IFN signature genes may potentially influence disease susceptibility through *cis-*regulatory mechanisms. We would, however, expect to identify many *cis*-eQTLs in these regions regardless of whether they associate with disease susceptibility or not. Therefore, instead of performing *cis*-eQTL analyses for all of the 73 dysregulated IFN signature genes, we first sought to identify variants that showed a disease association of *P*_*assoc*_<0.05 for subsequent evaluation of their role in altering gene expression. Genetic associations with SS susceptibility for 2,163 SNPs in regions of the 73 differentially expressed genes were tested using a combined dataset (Dataset 1; Table 1) from genome-wide genotyping arrays consisting of 765 SS cases and 3,825 population controls of European ancestry. We identified suggestive associations (*P*_*assoc*_<1×10^−4^; this threshold was determined by Bonferroni correction for independent variants with *r*^2^<0.2) of genetic variants within the *OAS1* region (top association at rs10774671, *P*_*assoc*_ = 8.47×10^−5^), which is regulated by type I IFNs (S1 Table). Furthermore, we identified suggestive associations in *ARGN* (S1 Table) and observed nominal associations (1×10^−4^≤*P*_*assoc*_<0.05) with SS susceptibility in 42 additional regions (S1 Table).

**Table 1:** Composition of independent cohorts used in the genetic association analyses.

Genotyping array		Initial genetic association analysis (Dataset 1)				Replication Dataset (Dataset 2)	
		Dataset 1A		Dataset 1B			
		Illumina OMNI1-Quad arrays (>1M SNPs)		Illumina OmniExpress arrays (>700K SNPs)		Taqman assay	OMNI1-Quad arrays OmniExpress arrays
Sample size		Case	Control	Case	Control	Case	Control
	Before QC	438	3,917	384	3,315	622	3,502
	After QC	395	1,975^a^	370	1,850^a^	514	3,466
Anti-Ro/SSA in cases after QC		Positive: 429, Negative: 154, No info: 182				Positive: 352, Negative: 126, No info: 36	

^a^. Each SS case in the initial genetic association study was genetically matched to 5 controls prior to analysis. Dataset 1A and 1B were merged into Dataset 1 in the initial genetic association analysis

**Fig 3:**
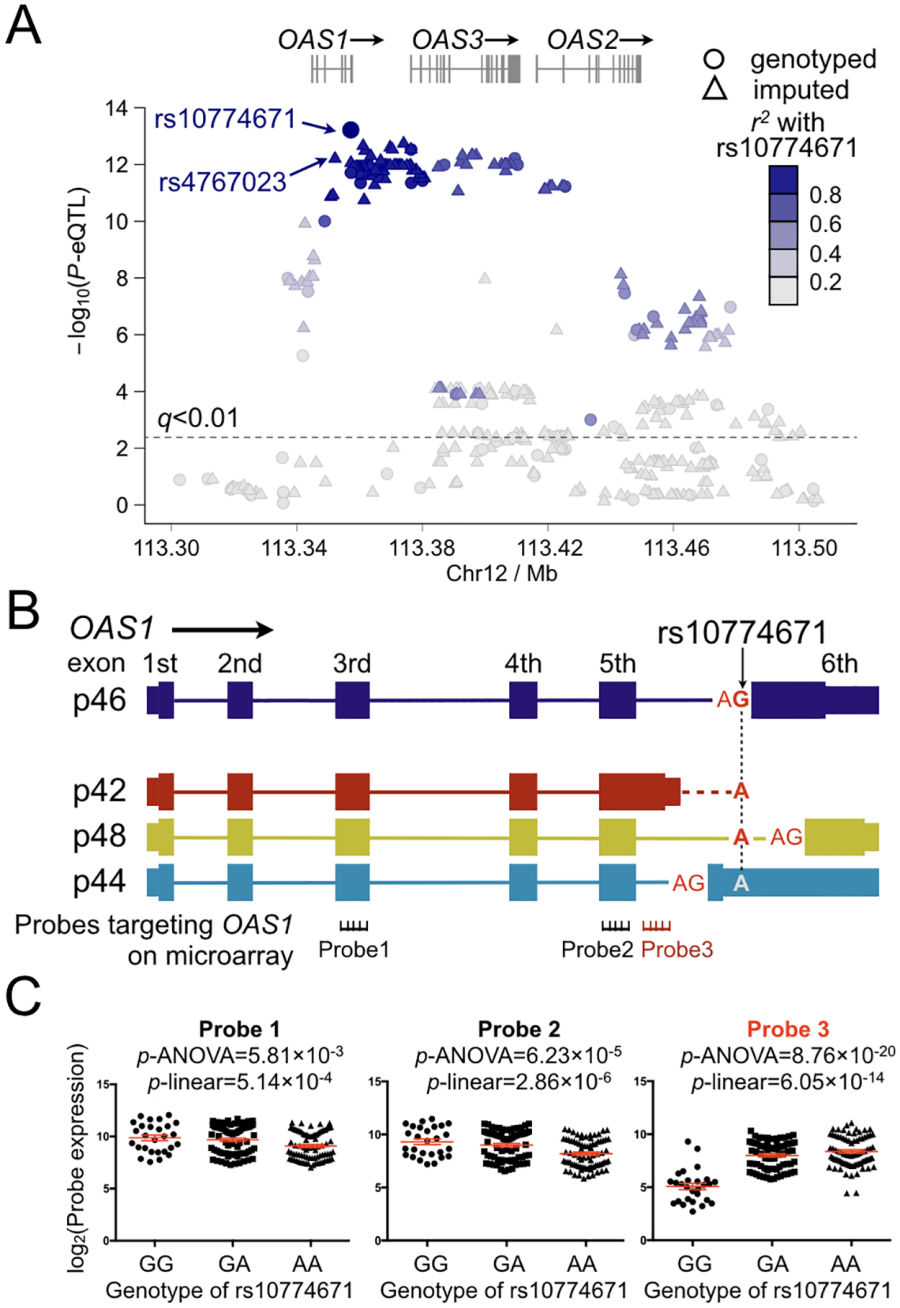
Results of *cis*-eQTL analysis in *OAS1* region. (A) After imputation, 453 variants near *OAS1* were tested for association with *OAS1* transcript expression using linear regression. The association of each variant with the transcript level of *OAS1* (represented by 3 probes on the microarray; see B) are plotted based on the most significant -log_10_(*P*_*eQTL*_) values. We identified *cis*-eQTLs within and near *OAS1*, with the top association at rs10774671 (*P*_*eQTL*_ = 6.05×10^−14^). The variant rs10774671 was also the most significant genotyped SS-associated SNP in the genetic association analysis (*P*_*assoc*_ = 8.47×10^−5^; The top imputed SS-associated variant rs4767023 is also marked on the plot). The *r*^2^ coded by colors indicating LD with rs10774671 are given in the figure. Variants above the dashed line were associated with *OAS1* transcript expression with *q*<0.01. No eQTL was observed for *OAS2* or *OAS3*. (B) The genomic structures of the isoforms of *OAS1* (p46: NM_016816; p42: NM_002534; p48: NM_001032409; and p44, as described previously and identified in our RNA-seq analysis) are shown. The location of rs10774671 and the splicing consensus sequence AG in p46, p48, and p44 are indicated. One probe on the microarray specifically detects the p42 isoform (Probe 3). (C) The *cis*-eQTL analysis was performed through integration of the microarray expression data of *OAS1* with the genotype data of rs10774671. The SS-associated risk allele A of rs10774671 was associated with higher expression level of the p42 isoform as determined by Probe 3. The A allele was associated with lower expression of total *OAS1* as measured by Probe 1 and Probe 2. The *cis*-eQTL analysis results were determined using both a linear model and ANOVA. The mean value and the standard error of the mean (Mean±SEM) in each group are plotted in red.

To determine whether these disease-associated genetic variants (*P*_*assoc*_<0.05) were related to the altered expression levels of their nearby differentially expressed IFN signature genes, we performed *cis*- and *trans-*eQTL analyses for all SNPs with *P*_*assoc*_<0.05 (173 SNPs in 44 regions; S1 Table) using a linear model by integrating the transcriptome and genotype datasets in 178 European individuals (108 anti-Ro/SSA positive SS cases, 55 anti-Ro/SSA negative SS cases, and 15 healthy controls). Variants within and near *OAS1* showed significant association with *OAS1* transcript expression (Fig 3A; S1 Table). In particular, three microarray probes targeting *OAS1* passed QC and were evaluated for *cis*-eQTLs (Fig 3B). The *OAS1* transcript levels measured by all of the three probes were found to be associated with nearby genetic variants (Fig 3C). The most significant *cis*-eQTL for all the three probes targeting *OAS1* was rs10774671 (*P*_*eQTL-Probe1*_ = 5.14×10^−4^, *P*_*eQTL-Probe2*_ = 2.86×10^−6^, and *P*_*eQTL-Probe3*_ = 6.05×10^−14^; Fig 3C). No eQTL was detected in any other differentially expressed genes (S1 Table). We also determined that none of these eQTL variants were associated with the two nearby genes, *OAS2* and *OAS3*. Additionally, no significant *trans*-eQTL was detected for *OAS1*. Therefore, we identified a variant associated with both SS susceptibility and gene expression in the IFN signature gene *OAS1*.

**Fig 4:**
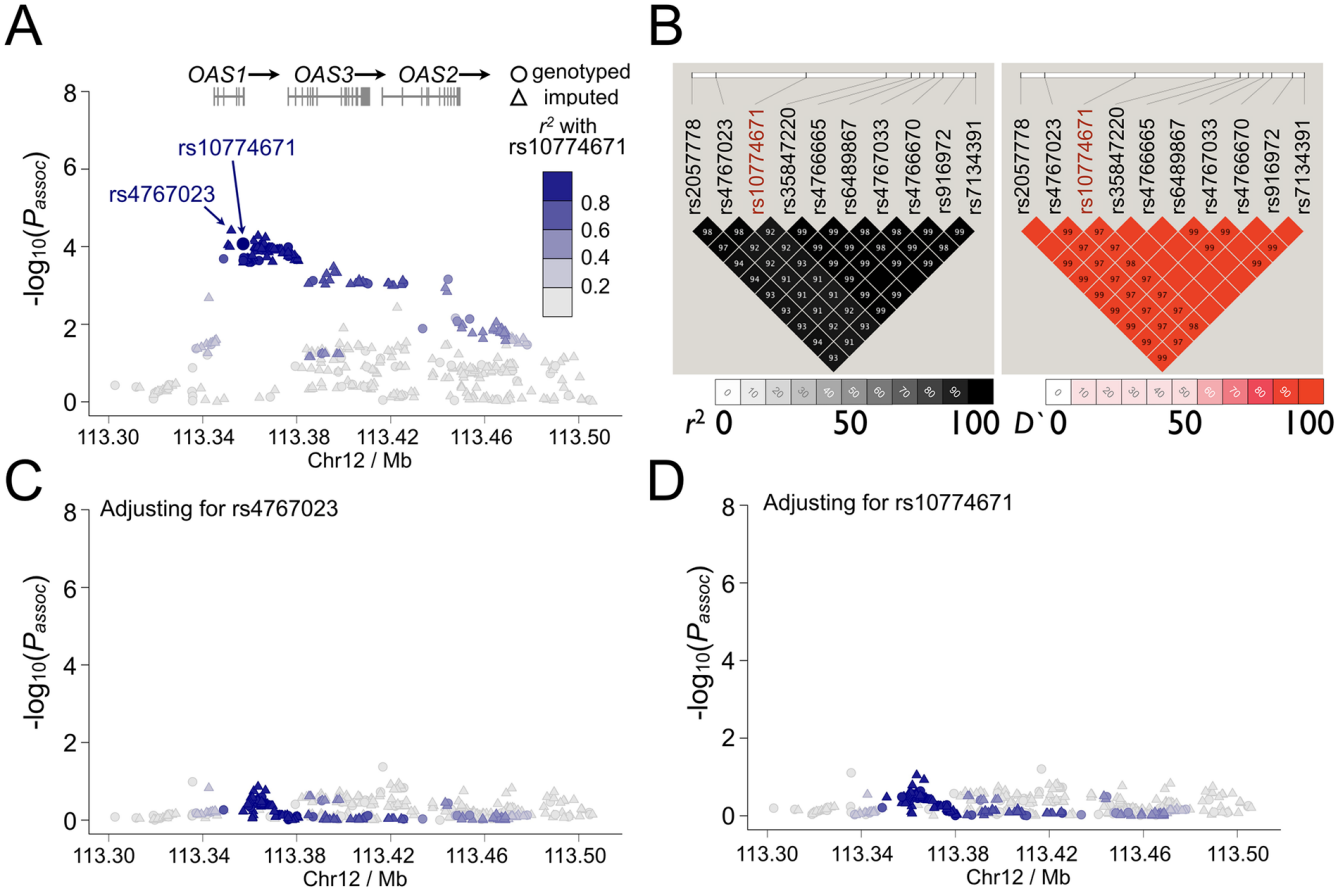
Results of genetic association analyses in *OAS1* region. (A) Genetic association analyses were performed using Dataset 1 (765 SS cases and 3,825 controls). The most significant association before and after imputation was at rs10774671 (*P*_*assoc*_ = 8.47×10^−5^) and rs4767023 (*P*_*assoc*_ = 3.82×10^−5^), respectively. (B) The LD structure of the *OAS1* region indicated by *r*^2^ and D' for SS-associated variants with *P*_*assoc*_<1×10^−4^ are shown. All these variants, including the top SS-associated genotyped SNP rs10774671, were in strong LD (*r*^2^>0.9). (C, D) In order to determine independency of the association signals observed in the *OAS1* region, we performed logistic regression analyses adjusting for the top SS-associated variants in the *OAS1* region. Adjusting for any variant with *P*_*assoc*_<1×10^−4^ could account for all other associations in the extended *OAS1* region (200kb). Examples shown here are conditional analyses adjusting for rs4767023 and rs10774671, respectively. The associations of each variant with SS were plotted based on -log_10_(*P*_*assoc*_) values. The *r*^2^ coded by colors indicating LD with the top SS-associated genotyped SNP rs10774671 are given in the figures.

To fine map this disease-associated region, imputation was then performed for the SS-associated *OAS1* region to increase the informativeness of the genetic association and eQTL analyses results. After imputation, the most significant association with SS in the *OAS1* region was at rs4767023 (*P*_*assoc*_ = 3.82×10^−5^; *r*^2^ = 0.98 with the most significant genotyped SNP rs10774671; Fig 4A), whereas the top eQTL remains at rs10774671 (Fig 3A). All the variants with *P*_*assoc*_<1×10^−4^ in the *OAS1* region were strongly correlated to each other (*r*^2^>0.9; Fig 4B) and could explain the association of the whole region through conditional analyses (Fig 4C and 4D). The top SS-associated variants and *cis*-eQTLs in the *OAS1* region, including rs10774671, were in strong LD (*r*^2^>0.9; Fig 4B), thus challenging the selection of potentially functional variant(s) based on results from the association analyses. However, the top eQTL variant, rs10774671, is an A/G substitution within the consensus sequence of a splice acceptor site at the junction of the 5^th^ intron and the 6^th^ exon of *OAS1* (Fig 3B), and is known to alter normal splicing and induce isoform switching of *OAS1* [51]. In addition, all other SS-associated variants (*P*_*assoc*_<1×10^−4^) in the *OAS1* locus were either intronic or outside of coding regions, lacking functional genomic elements mapped to the SNP as determined by the ENCODE project [48, 52]. Also, we performed a co-localization analysis using eCAVIAR [53] to identify the potential causal variant in the *OAS1* region. We estimated colocalization posterior probability (CLPP) scores for all the tested 453 variants, and rs10774671 has the highest CLPP score among all the variants (S2 Table). Therefore, we prioritized this SS-associated *cis*-eQTL variant, rs10774671, for further replication and functional studies.

We replicated the genetic association of rs10774671 with SS susceptibility in an independent dataset (Dataset 2; Table 1) consisting of 514 European SS cases and 3,466 European population controls (genotyped using TaqMan assays, *P*_*rep*_ = 5.16×10^−6^; odds ratio = 0.71; 95% confidence interval = 0.63–0.83). Meta-analysis was performed to combine the results between the initial genetic association study (Dataset 1) and the replication cohorts (Dataset 2) and established the association of rs10774671 with SS risk (*P*_*meta*_ = 2.59×10^−9^; odds ratio = 0.75; 95% confidence interval = 0.66–0.86; risk allele [the A allele] frequency: case = 0.70, control = 0.64; with no heterogeneity between the two datasets as determined by *I*^2^ = 0). We also performed a stratified analysis and a permutation analysis using merged samples from Dataset 1 and Dataset 2 to determine whether the observed genetic association was restricted to anti-Ro/SSA positive or negative patients. We did not find any evidence to support the genetic effect to be specific to any sub-group of the patients (S1 Fig). In summary, we identified a potential causal variant, rs10774671, that was associated with SS susceptilibty, likely through its impact on the expression of a key IFN signature gene, *OAS1*.

### Impact of rs10774671 on *OAS1* splicing

Following establishment of the association between rs10774671 and SS susceptibility, we further determined the influence of different genotypes on the alternative splicing of *OAS1*. Four isoforms of *OAS1* are annotated in the NCBI Reference Sequence (RefSeq; http://www.ncbi.nlm.nih.gov/refseq) database, of which we analyzed p46, p42, and p48, and p44, an un-annotated isoform previously reported in RNA-sequencing (RNA-seq) studies [54–56] (Fig 3B). The difference between these isoforms is confined to their 3' end where rs10774671 influences alternative splicing, yielding amino acid sequences of different lengths and composition. In the microarray experiments, one probe targeting *OAS1* specifically recognized the 3' end of the p42 isoform (Fig 3B). The risk allele A of rs10774671 was correlated with higher expression levels of p42 (Fig 3C, right panel). However, we were not able to determine the influence of rs10774671 on the expression of other isoforms due to lack of isoform-specific probes on the microarray.

**Fig 5:**
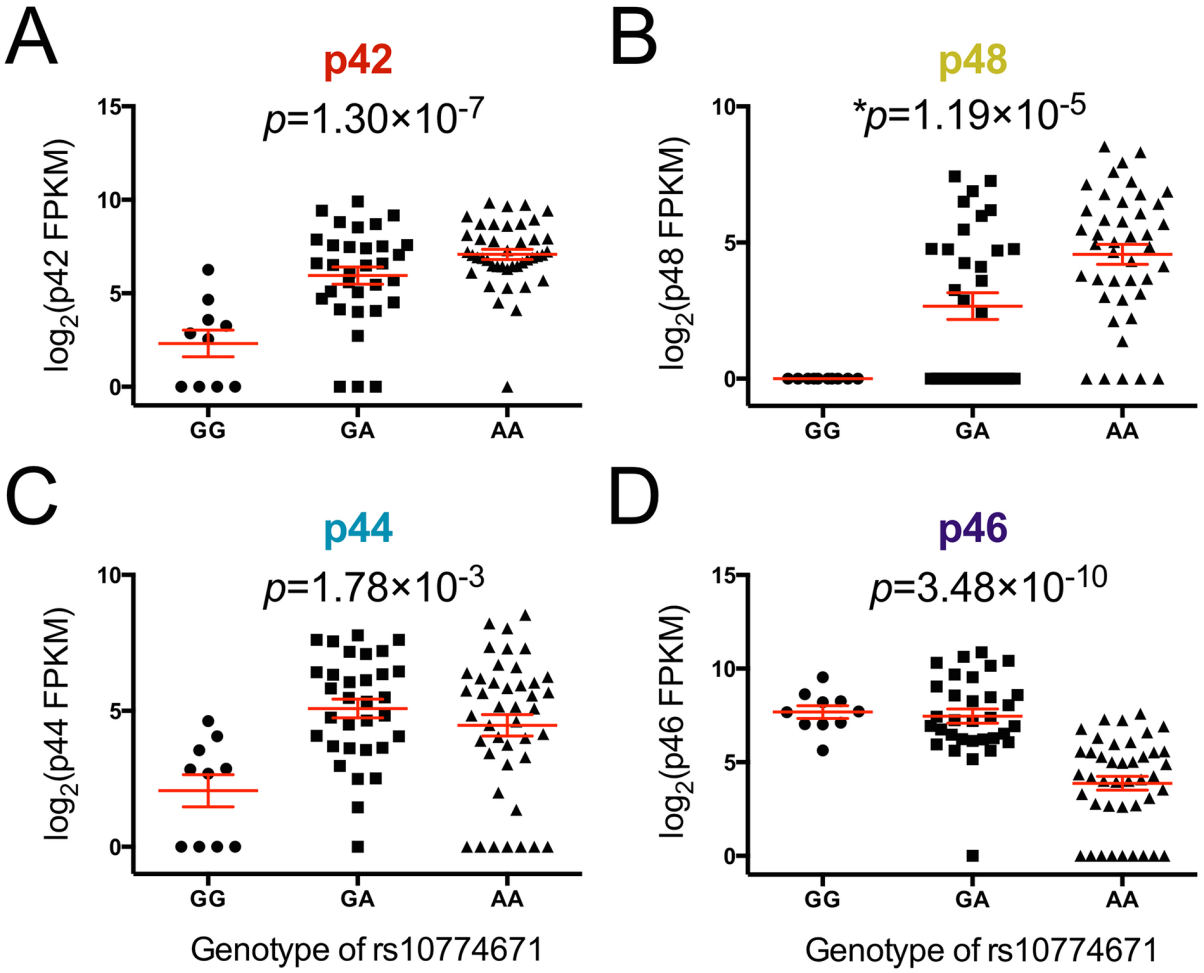
Correlation of rs10774671 with expression levels of each *OAS1* isoform. The expression level of each isoform was determined using RNA-seq. The abundance of each isoform was measured by FPKM in Cufflinks and was compared between individuals with different genotypes of rs10774671. The risk allele A of the SS-associated variant rs10774671 was correlated with higher transcript expression of (A) p42, (B) p48, and (C) p44 isoforms, but lower levels of the (D) p46 isoform. The *P* value for each analysis was determined using one-way ANOVA except for p48 (as all samples in the GG group have zero values, Kruskal-Wallis test was used for p48, not assuming equal standard deviation across groups). The mean value and standard error of the mean of each group are plotted in red. The results were replicated using quantitative real-time PCR with primer sets targeting specific *OAS1* isoforms (S4 Fig; S3 Table).

In order to determine the influence of rs10774671 on the expression of each alternatively spliced isoform of *OAS1* and compare *OAS1* isoform composition, we performed RNA-seq on whole blood from 57 SS cases and 27 healthy controls. After QC, the reads were aligned to the human genome using TopHat [57] without gene annotation to facilitate the detection of potentially novel isoforms of *OAS1*. The transcript level of each isoform was compared across samples with different genotypes of rs10774671 based on the measurement of fragments per kilobase of transcript per million mapped reads (FPKM) using Cufflinks [58]. Consistent with our microarray results, the SS risk allele A of rs10774671 was correlated with higher expression levels of p42 (*P* = 1.30×10^−7^; Fig 5A). Increased production of other alternatively spliced isoforms of OAS1, including p48 and p44, was also observed in subjects with the SS risk genotypes (GA and AA) of rs10774671 (Fig 5B and 5C). In contrast, transcript levels of the p46 isoform, was decreased in samples with the A allele (*P* = 3.48×10^−10^; Fig 5D), consistent with previous reports that interruption of the splicing consensus sequence inhibit formation of the p46 isoform [56]. These results were further confirmed by quantitative real-time PCR using primer sets targeting the specific *OAS1* isoforms (S2 Fig; S3 Table). Therefore, we found that the SS-associated variant rs10774671 is a functional variant that influences alternative splicing of *OAS1*.

Since *OAS1* is part of the IFN signature and its expression levels are correlated with the autoantibody status, we also performed a stratified eQTL analysis to investigate whether the eQTL effects are specific to any sub-group of the SS patients based on their anti-Ro/SSA positivity. We stratified the SS case samples into anti-Ro/SSA positive patients (n = 27) and anti-Ro/SSA negative patients (n = 30), and performed eQTL analyses on each of the *OAS1* isoforms using linear regression while adjusting for sex. Despite reduced statistical power, we identified significant eQTL results for the p46, p42, and p48 isoforms in both subsets of samples. By using the *Z*-test as described in S1 Fig, we did not find any significant difference of the eQTL effects between the two sub-groups (S3 Fig).

**Fig 6:**
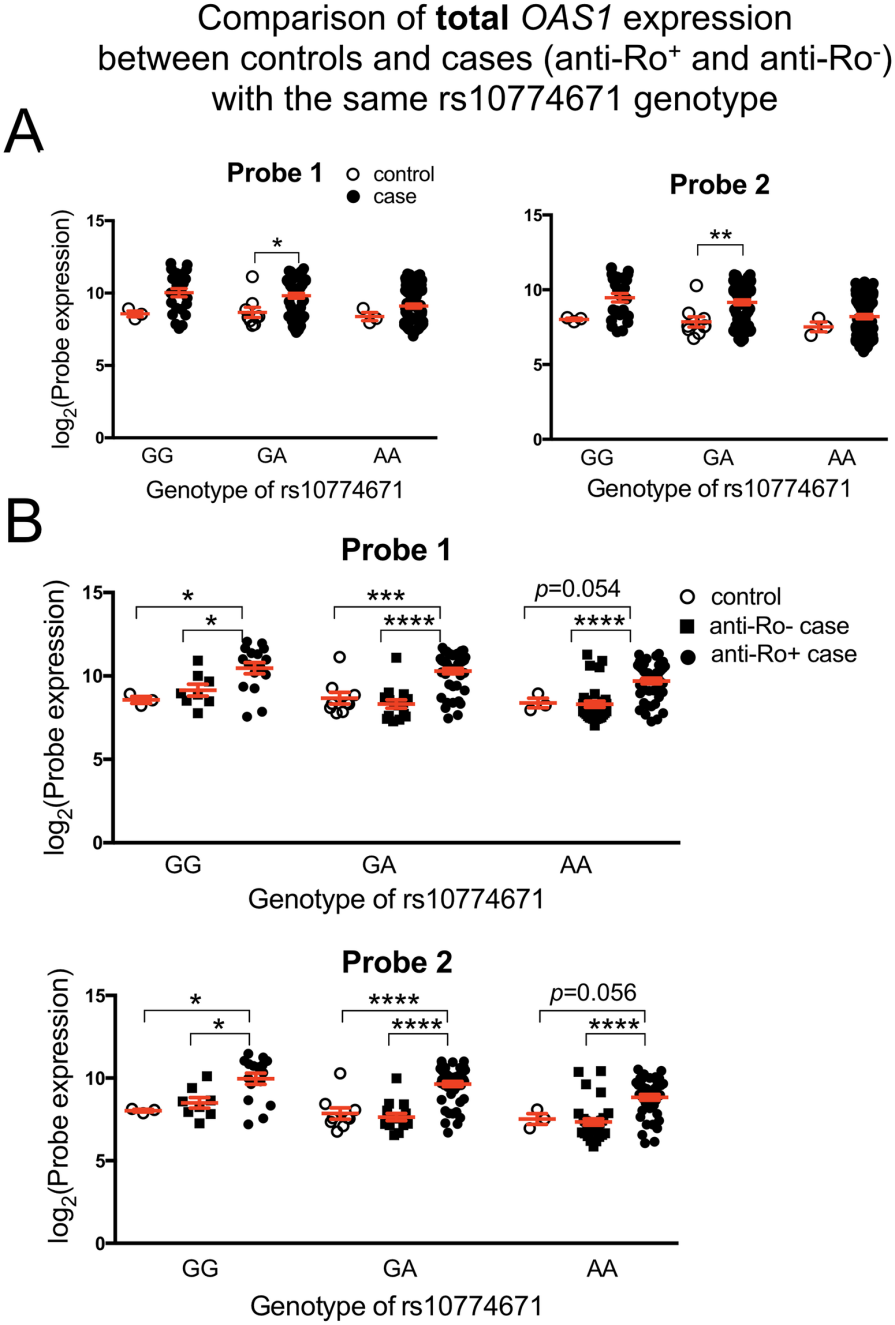
Correlation of rs10774671 genotype with total *OAS1* transcript levels as determined by microarray. Two probes targeting total *OAS1* transcript expression passed QC on the microarray (Probe 1 and Probe 2; Fig 4B). (A) Statistically significant differences were observed for the total *OAS1* transcript levels between the case and control groups carrying the same genotype (GA). (B) When SS case subjects were further divided by anti-Ro/SSA status, the overall expression of *OAS1* transcripts was significantly higher in anti-Ro/SSA positive SS patients compared to either controls or anti-Ro/SSA negative cases within the same genotype group. *P* values were determined using two-tailed *t* test (Significance level: * *P*<0.05; **** *P*<0.0001). The Mean±SEM of each group are plotted in red.

Comparing the total *OAS1* transcript level from the microarray study within each genotype revealed significantly higher gene expression in SS patients as compared to control in the GA group (Fig 6A). There is a trend towards higher total *OAS1* transcripts in the AA and GG groups of SS patients as well, though it is not statistically significant. Interestingly, the highest transcript levels are seen in the anti-Ro/SSA positive cases (Fig 6B), significantly higher than both anti-Ro/SSA negative cases and healthy controls. The same results were observed in the RNA-seq data (S4 Fig), indicating that the total *OAS1* transcript levels regardless of isoform are also influenced by disease status or the presence of autoantibodies.

### Functional characterizations of *OAS1* isoforms

**Fig 7:**
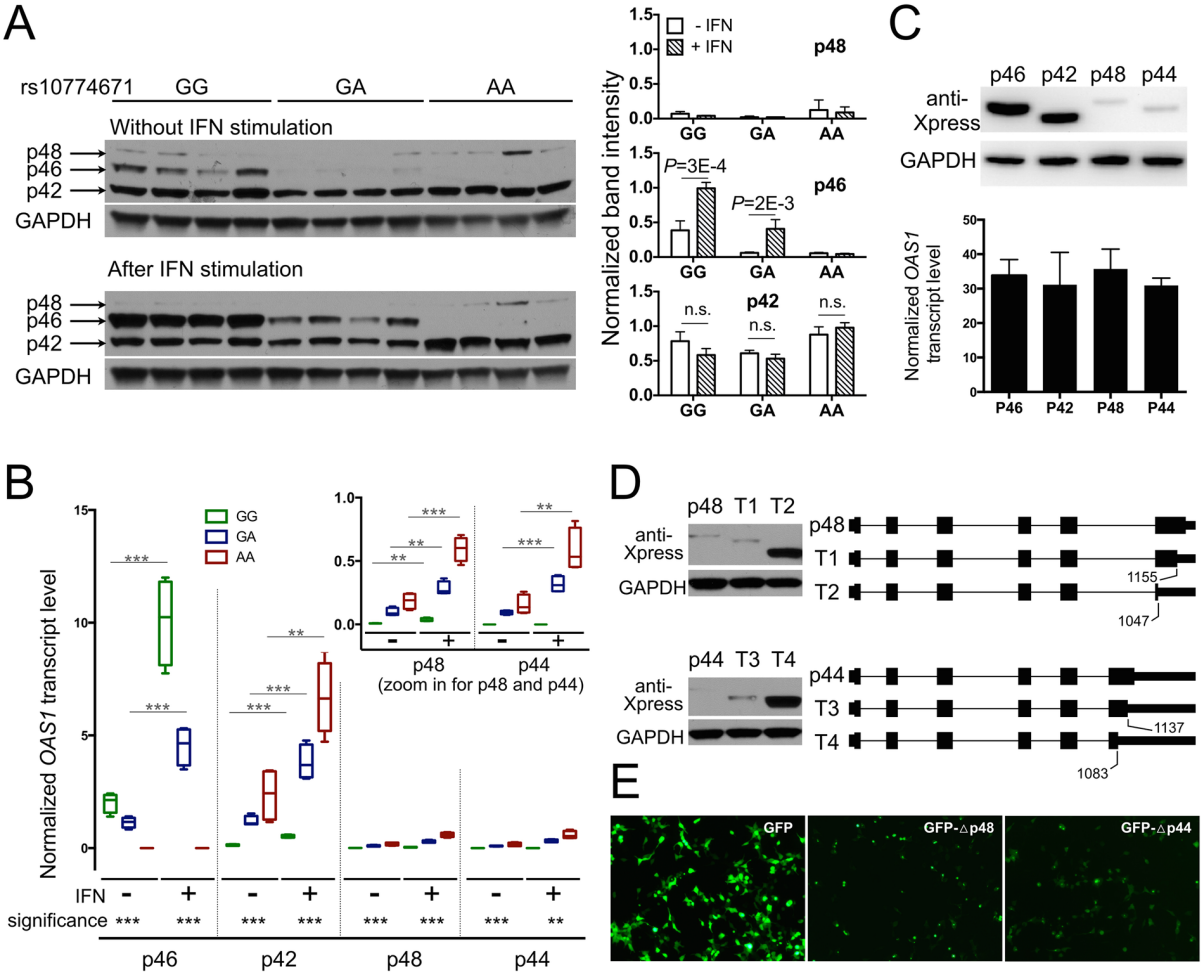
Functional characterizations of *OAS1* isoforms. (A) Protein expression of OAS1 isoforms was evaluated in EBV-transformed B cells from SS patients (four independent samples from each genotype group) using anti-OAS1 antibody targeting the shared epitope of all the isoforms. The stimulated cells were treated with universal type I IFN (1500U/ml) for 24hrs. The p44 isoform was not detectable using western-blot due to its low expression. The right panel shows quantified band intensity normalized to the GAPDH in each sample. (B) The transcript levels of each *OAS1* isoform from the same sets of cells described above were determined using real-time PCR. Consistent with the RNA-seq results, the SS-associated risk allele A of rs10774671 was correlated with decreased levels of p46 and increased expression of the p42, p48, and p44 isoforms (significance levels are shown at the bottom). The transcript levels of all the isoforms significantly increased after IFN stimulation (two-tailed *t* test); however, only p46 had increased protein production after IFN stimulation. (Significance level: ** *P*<0.01; *** *P*<0.001) (C) Individual isoforms of *OAS1* tagged with Xpress epitope were cloned and transfected into HEK 293T cells for 48hrs. The p48 and p44 isoforms had impaired protein expression compared to p46 and p42, although their transcript levels were equivalent as determined by real-time PCR (n = 4; normalized to *HMBS*). (D) The full-length and truncated *OAS1* p48 and p44 isoforms were cloned into HEK 293T cells. Western-blot indicated the lack of expression of the full-length p48 and p44 isoforms, whereas the truncation of both isoform transcripts (T2 and T4) was able to restore protein expression. (E) The 3' alternatively spliced terminus of different *OAS1* isoforms were linked to the 3'-end of GFP to observe their influence on GFP protein expression in HEK 293T cells. The 3'-terminus from the p48 and p44 isoforms resulted in decreased expression of GFP.

To further dissect the functional mechanism of rs10774671 in predisposing disease risk, we utilized Western blots to evaluate the difference in protein levels of the normally spliced isoform p46 (formed by the protective allele G of rs10774671) and the alternatively spliced isoforms of OAS1 in EBV-immortalized B cells from SS patients. Consistent with the RNA-seq results, the protein expression of p46 was substantially lower in subjects carrying the A allele of rs10774671, whereas p42 was the dominant isoform in the GA and AA subjects without stimulation (Fig 7A). Interestingly, both protein and mRNA levels of the p46 isoform were upregulated after stimulation by type I IFN in the GG and GA subjects (*P* = 3×10^−4^ and *P* = 2×10^−3^, respectively; Fig 7A and 7B). However, protein expression of the p42 isoform remained unchanged upon IFN stimulation (Fig 7A), even though its transcript level significantly increased after stimulation (Fig 7B). The protein expressions of p48 and p44 were low in all of the samples, and not responsive to type I IFN stimulation (Fig 7A). We then cloned and transfected each isoform into human embryonic kidney (HEK) cell line 293T cells and observed similar protein expression results as in EBV cells: lower protein levels for p48 and p44 compared to p46, even though their transcript levels were equivalent (Fig 7C). These results suggest that the alternative isoforms of *OAS1* that are associated with the disease risk variant of rs10774671 fail to generate proteins after transcription.

The catalytic OAS1 domain is located at the N terminus, though the isoforms differ in their C terminus. It has been suggested that these differences affect affinity of OAS1 protein for different viruses [59]. However, our data suggested that the alternatively spliced 3'-terminus influenced the lack of post-transcriptional expression of the p48 and p44 isoforms. To test this hypothesis, we generated several truncated forms of p48 and p44 at the 3'-terminus and transfected them into HEK 293T cells. The truncation of both p48 and p44 transcripts at the 3'-end resulted in restoration of protein expression (Fig 7D). Our results demonstrated that the alternatively spliced 3'-end between 1,047 and 1,155 bp of the p48 isoform and the 3'-end between 1,083 and 1,137 bp of the p44 isoform were responsible for the impaired protein expression (Fig 7D). In addition, we recombined the green fluorescent protein (GFP) transcript with the 3'-end from different *OAS1* isoforms and expressed them in HEK 293T cells. The alternatively spliced 3'-terminus of p48 and p44 resulted in reduced expression of GFP when linked to the 3' end of GFP transcript (Fig 7E; S5 Fig). These results further confirmed the impact of the alternatively spliced 3'-end of *OAS1* on protein expression. Determining mechanisms for how the 3'-terminus from the alternatively spliced *OAS1* isoforms influences protein expression and type I IFN responsiveness needs further study.

## Discussion

Overexpression of genes in the IFN pathway is a distinctive feature of multiple autoimmune diseases, though no evident mechanism has thus far been revealed. We identified and established rs10774671 as a risk locus for SS. The A allele of rs10774671 is correlated with reduced OAS1 enzymatic activity in human peripheral blood mononuclear cells [51], and is associated with increased susceptibility to West Nile virus [60] and chronic hepatitis C virus infections [61]. OAS1 is a member of the 2'-5'-oligoadenylate synthetase family, which is upregulated by type I IFNs during innate immune responses to viral infection and activates latent RNase L, leading to viral RNA degradation and clearance [62, 63]. The SS risk allele A of rs10774671 causes alternative transcript splicing and consequently less functional isoforms are activated by type I IFNs.

Failure to clear virus might lead to subclinical, chronic infection that drives the sustained overexpression of IFN, but viral proteins may also indirectly cause IFN production through adaptive immune responses. For example, antibodies generated towards EBV nuclear antigen-1 cross-react with Ro/SSA [21], and anti-Ro/SSA antibodies may in turn form immune complexes that stimulate type I IFNs [64]. As viruses evidently play a role in SS pathophysiology [18], genetic variants affecting the antiviral properties of OAS1 might be a contributing factor. A recent study showed that while the presence of antibodies to hepatitis D virus was equal in SS patients and otherwise healthy controls, the virus itself was present in significantly more patients [65], indeed suggesting that viral clearance is restrained. Epitope spreading [66, 67], antibody cross-reaction [21], or molecular mimicry [68, 69] are likely consequences of subclinical, chronic or recurrent infection.

The basal activity of OAS1, which varies greatly among individuals, is thought to be under strong genetic control [51]. The enzyme activity in the GG genotype with predominantly the p46 isoform is higher than GA (intermediate) and AA (low) [51]. OAS1 isoforms p42 and p46 have been detected at the protein level in human cells, whereas the p44a/p44b, p48, and p52 isoforms have been detected at mRNA levels [54, 70–72]. In addition to the RNase L activation properties, the tetramer forming p48 isozyme also exhibits proapoptotic activity [73], a property partly accredited to IFN-γ [74], and is shown to interact with Bcl-2 [75]. Bcl-2 is an anti-apoptotic protein negatively regulated by Ro52 [76], and in salivary gland epithelial cells Bcl-2 is essential in regulation of IFN-γ induced apoptosis [77]. It has been postulated that p46 is a more efficient synthetase than p48, explaining the increased basal activity of p46 [51]. Although there is no evidence showing that the differences in the C-terminus alter protein function [78], our truncation experiments indicated that the alternatively spliced C-terminus governs the post-transcriptional protein expression.

Interestingly, we found lower protein expressions of p44 and p48 after type I IFN stimulation despite equivalent transcript levels compared to p46. This indicates that p44 and p48 production, which is governed by the A allele, is less responsive to IFN stimulation as compared to p46. Lack of response to IFNs has also been seen in multiple sclerosis (MS), in which patients carrying the homozygous rs10774671 GG genotype, a protective genotype in MS associated with less active disease, were more responsive to IFN-β treatment than AA and AG patients, as measured by time to first relapse [79].

We searched the Genotype-Tissue Expression (GTEx) database and confirmed the association between rs10774671 and *OAS1* expression in whole blood [80]. We also searched eQTL for all our top variants in the 43 SS-associated genes (besides *OAS1*) as well as any variants in LD with those top variants (*r*^*2*^>0.8). Out of the 614 variants we checked, two variants were also eQTLs for their corresponding genes: the top SS-associated variant in *ANKRD22* (rs1147601, *P*_*assoc*_ = 2.38×10^−3^, *P*_*eQTL-GTEx*_ = 6.4×10^−6^) and the top SS-associated variant in *EPSTI1* (rs7323736, *P*_*assoc*_ = 1.79×10^−2^, *P*_*eQTL-GTEx*_ = 2.6×10^−6^). However, both of these variants were only nominally associated with SS susceptibility and did not pass our suggestive significance threshold for disease association (*P*_*assoc*_<1×10^−4^). Nevertheless, these variants and genes could be plausible targets for future replication studies to assess their disease associations.

The rs10774671 A/G variant is a common splice site variation, and there is a skewed distribution of genotypes in autoimmune diseases like type I diabetes (T1D) [81] and MS [79] despite ambiguous genetic association with disease: the alternative allele A renders risk to SS and MS, whereas the reference allele G increases susceptibility to T1D. We hypothesized that these opposite risk effects may be due to different functional isoform usages in different disease-relevant tissues. Through searching the GTEx database for rs10774671 eQTLs, we found rs10774671 is a significant eQTL of *OAS1* in 5 tissues (S6A Fig). Interestingly, the eQTL effect in the Esophagus—Mucosa tissue is in the opposite direction compared to other tissues. In whole blood, the p46 isoform is predominant, thus the A allele caused reduced expression of *OAS1* as a whole (S6B Fig); however, in Esophagus Mucosa, the isoform is p42 (S6C Fig) and results in an opposite effect of rs10774671 on the total *OAS1* expression. We propose that the ambiguous genetic effects of rs10774671 on different diseases might be due to different functional isoforms in disease-relevant tissues (not necessarily Esophagus—Mucosa). While reduced expression of the functional isoform p46 in whole blood increases risk of SS and MS, it protects individuals from T1D. The downstream differences of various isoforms in protein levels, isoform expression, responsiveness to IFN, and basal activity between genotypes flag OAS1 as a highly relevant protein in autoimmune diseases, despite no direct effect on IFN expression.

*OAS1* is one of several genes relevant in overall IFN response found to be disease associated in SS. Others include *IL-12A* [15], which can induce both type I and type II IFNs [82]; *STAT4* [15], which, although not explicitly overexpressed in the IFN signature, plays an important role in the cross-talk between type I and type II IFNs [83–85]; and *IRF5* [15], a transcription factor in the IFN pathway [86]. The rs10774671 is a known *cis*-eQTL and splicing QTL, observed in whole blood [87] as in our study, in lymphoblastoid cells [88], and in monocytes, both naïve CD14 and in cells stimulated with LPS and IFN-γ [89]; but no *trans*-eQTLs are known. It is possible that other variant(s) in high LD with rs10774671 could contribute additional functional impact(s), such as the rs11352835 in exon 7 seen in MS [59]. Genomic editing approaches that introduce single point mutations or deletions in the *OAS1* region will further advance the dissection of the causal SS-associated variant in this haplotype. Animal models that express the risk isoforms due to the risk allele can also be used to observe whether they spontaneously develop SS-like symptoms, and whether the chances for developing such symptoms increase after exposure to viral infections. Our study also highlights the importance of utilizing genomic convergence to identify and prioritize susceptibility genes for human complex disease. The complex mechanisms underlying the IFN signature in SS cannot be explained as a single eQTL driven overexpression. However, we have in this study established *OAS1* as a risk locus with functional consequences affecting isoform composition, and that may play a fundamental role in dysregulation of both viral clearance and apoptosis.

## Materials and methods

### Subjects

All patients in this study fulfilled the 2002 American-European Consensus Group (AECG) criteria for primary SS [7]. Seropositivity of anti-Ro/SSA autoantibodies was determined by the antibody index ≥1 using the Bio-Plex assay (Bio-Rad) following the manufacturer’s protocol. The present study was approved by the Oklahoma Medical Research Foundation Institutional Review Board (IRB#1—Biomedical), operation under Federalwide Assurance (FWA) # 00001389 and IRB # 00000114 under IORG 0000079 approved by the Office for Human Research protection (OHRP), Department of Health and Human Services (DHHS). The OMRF IRB is in compliance with local regulations and the regulations of the United States Food and Drug Administration as described in 21 CFR Parts 50, 56 and 11, the International Conference on Harmonization (ICH) E6, and the United States Department of Health and Human Services at 45 CFR 46. The current study was approved under IRB#07–12 and all patients provided written informed consent.

#### Transcriptome profiling study

SS patients were evaluated by expert clinicians at the University of Minnesota or the Oklahoma Medical Research Foundation (OMRF) as described previously [90]. Samples subjected to microarray-based transcriptome measurements included 182 patients with SS and 76 healthy controls. After QC assessments (see below), 115 anti-Ro/SSA positive SS cases and 56 healthy controls were included in the transcriptome profiling analysis. We assessed the distribution of the five main nucleated blood cell subpopulations (granulocytes, lymphocytes, monocytes, basophils and eosinophils) derived from complete blood cell counts from SS patients and healthy controls to evaluate whether differences in their proportions might contribute to differential expression of transcriptional signatures (S7 Fig). A total of 178 European subjects (108 anti-Ro/SSA positive SS cases, 55 anti-Ro/SSA negative SS cases, and 15 healthy controls) for whom genotype data were also available were used in the *cis*- eQTL analysis.

#### Genetic association study

All SS cases used in the genetic association analyses were collected through the Sjögren’s Genetics Network and organized at the OMRF. Two datasets (Dataset 1A and 1B) were combined for the initial genetic association analysis (Table 1). All subjects in Dataset 1A have been previously described in a genome-wide association study [15]. In Dataset 1B, 384 SS cases of European descent were genotyped and subjected to QC measurements outlined below. The genotype data of 3,315 population controls in Dataset 1B were obtained from the database of Genotypes and Phenotypes (dbGaP; http://www.ncbi.nlm.nih.gov/gap). Each SS case was genetically matched to five population controls in Dataset 1A and 1B, respectively, using the identity-by-state (IBS) to assess allele sharing as implemented in PLINK v1.07 [91]. The remaining controls were used in the replication study (Dataset 2). A total of 622 SS cases and 3,502 population controls were subjected to QC in the replication phase (Dataset 2; Table 1).

#### RNA-seq study

For RNA-seq experiments, a total of 90 European subjects were evaluated, including 27 anti-Ro/SSA positive SS cases, 33 anti-Ro/SSA negative SS cases, and 30 healthy controls.

### Microarray experiments and analyses

#### RNA processing and measurements

Total RNA was obtained by blood collection into PAXGene tubes (BD Company) and extracted following manufacturer’s protocols (Qiagen). Excess globin transcripts were removed using GLOBINclearTM (Ambion). RNA concentrations were determined using a NanoDrop spectrophotometer (Thermo Scientific) based on Optical Density values at A260. RNA quality was assessed by Agilent 2100 Bioanalyzer based on 28S/18S ribosomal RNA ratio and RNA integrity number. Double stranded cDNA was synthesized using a T7 promoter, and biotin-labeled cRNA was transcribed using the Illumina TotalPrep RNA Amplification System (Ambion). Samples were hybridized to Human WG-6 v3.0 BeadChip microarrays (Illumina) containing 48,803 mRNA probes in 37,805 unique genes per array. Microarrays were washed under high stringency and labeled with streptavidin-Cy3, and fluorescent intensity-based gene expression data were collected using Illumina’s BeadStation 500 scanner or iScan.

#### QC and statistical analyses

Unless otherwise stated, all statistical analyses were performed in the R Bioconductor suite. Microarray experiments were performed in two batches, with 93 SS cases and 34 healthy controls in Batch 1 and 89 cases and 42 controls in Batch 2. Raw intensity values for the two datasets were background subtracted separately using Illumina BeadStudio software. Identification of outlier and poor-performing samples was accomplished by applying the package arrayQualityMetrics (AQM) [92] to log_2_-transformed microarray data from each experiment. QC measures were applied to each dataset to filter out transcripts expressed in <10% of the subjects (detection call threshold *P*<0.05) and probes with differential missingness rates (*P*<0.001 by Fisher’s exact test) between the two datasets. The remaining probes were then compared against data tables from the Re-annotation and Mapping of Oligonucleotide Array Technologies (ReMOAT) [93], in which Illumina BeadArray probe quality was extensively assessed and re-annotated. Each dataset was then independently normalized using Robust Multiarray Average (RMA) [94], followed by log_2_ transformation and quantile normalization. The ComBat program was subsequently applied to the combined dataset to adjust for non-biological experimental variation (i.e. batch effects) [95]. Final re-annotation of un-annotated probes was performed manually using the NCBI probe database (http://www.ncbi.nlm.nih.gov/probe) and UCSC Genome Browser alignment (https://genome.ucsc.edu) [52]. Probes mapping to un-annotated genes or having inconsistent results from the two databases were removed from the analysis. The results and pipeline of the QC and normalization procedures applied to microarray data are summarized in (S8 Fig). Gene expression comparisons were performed using Welch’s *t*-test between the mean expression values in SS cases and controls. The clustering of genes and samples was performed in Cluster 3.0 [96] and visualized in Java TreeView [97]. Unless otherwise noted, unsupervised hierarchical clustering was performed using centroid linkage with uncentered correlation for both genes and samples.

### Genetic association experiments and analyses

#### Genotyping and imputation

Genotyping for Dataset 1A was described previously [15]. Genotypes for Dataset 1B were obtained using the Illumina OmniExpress arrays at OMRF following the manufacturer’s protocol. To increase informativeness, imputation was conducted in subjects from the merged datasets (Dataset 1) for the *OAS1* region (chr12: 113294739–113507712; hg19) meeting the criteria for suggestive association with SS (*P*_*assoc*_<1×10^−4^). Imputation was performed using IMPUTE2 and the European Impute2 1000 Genomes Phase 1 April 2012 reference panel [98–100]. A probability threshold of 0.9 and information score of >0.5 were applied to the imputed genotypes in addition to the QC criteria described below for the association analyses. Genotypes for the replication study (Dataset 2) were determined using TaqMan probes and reagents (Life Technologies) following the manufacturer’s protocol.

#### QC process

Variants and samples in the genetic association analysis were subjected to a strict QC procedure as described previously [15]: single-nucleotide polymorphism (SNP) call rate >95% in all individuals; minor allele frequency >1%; Hardy-Weinberg proportion test with a *P*>0.001 in controls; and *P*>0.001 for differential missingness between cases and controls. Samples from Dataset 1A and Dataset 1B passing QC were retained if results showed: >95% call rate for all variants; no excessive increased heterozygosity (>5 standard deviations from the mean); and no relatedness determined by identity-by-descent (IBD) >0.4 using PLINK v1.07 [91]. Population substructure was identified using EIGENSTRAT [101] with independent genetic markers (*r*^2^<0.2 between variants). The resulting Eigenvectors were used to distinguish the four continental ancestral populations with the following HapMap samples: Africans (ASW, LWK, MKK, and YRI), Europeans (CEU and TSI), Hispanic and East Indians (MEX and GIH), and Asians (CHB, CHD, and JPT) [102, 103]. The first two principal components (PCs) output by EIGENSTRAT were plotted and used to identify samples outside the European cluster [101, 102] (S9 Fig). Outliers from the European population were removed from further analysis. Population stratification analysis was performed for the replication cohort using genotype data from a previous study for the 622 SS cases [15] and genotype data from the respective dbGaP studies.

#### Statistical analysis

A logistic regression model was used to test the association of genetic variants with SS susceptibility in PLINK v1.07 [91]. The additive genetic model was calculated for variants within and 50kb flanking the 73 differentially expressed transcripts while adjusting for gender and the first three PCs as determined by the Scree test to evaluate the loading of each PC for the amount of variance explained [104]. Meta-analysis of rs10774671 between the initial genetic association analysis (Dataset 1) and the replication study (Dataset 2) were calculated using a *Z-*score weighted by the sample size of each dataset in METAL [105]. We tested our logistic regression model for deviation from additivity using PLINK in both the discovery dataset and replication dataset, and neither model shows significant deviation (*p* = 0.40 and *p* = 0.31, respectively). Logistic regression adjusting for the most significant variants (conditional analysis) was performed to determine independence of the association. LD and probable haplotypes were determined using Haploview [106].

### *Cis*- and *trans-*eQTL analysis

Quantitative levels of the differentially expressed transcripts from the microarray analysis were used as phenotypic traits in 178 European subjects described above. Variants showing nominal association with SS (*P*_*assoc*_<0.05) in the genetic association analysis were selected to test for *cis*-eQTLs, defined by variant-transcript pairs within 50kb of the target genes or *trans*-eQTLs for variants at least 1Mb away. Association of genotype with transcript expression was evaluated using both linear regression (adjusted for gender and disease status) and analysis of variance (ANOVA) in Matrix-eQTL [107]. FDR-adjusted *P* values were calculated to determine the significance of the eQTL. The results of the *cis*-eQTL analyses were plotted in Prism 6. We also used a tool, PEER, based on a Bayesian framework to adjust for unknown non-genetic factors in gene expression [108]. We transformed our expression values using all the genes that passed QC by running PEER for 15 factors. We then used the PEER residuals from the 44 SS-associated (*P*_*assoc*_<0.05) IFN signature genes as quantitative traits to determine eQTL while adjusting for other known potentially confounding factors: sex, disease status, anti-Ro/SSA status, and age.

In addition to additive genetic models, we also performed eQTL analyses using linear regression by three other models: recessive (recode genotype from 0,1,2 [where 2 equals to AA] to 0,0,1), dominant (0,1,1), and overdominant (0,1,0). We used the coefficient of determination (*R*^2^) to evaluate the goodness-of-fit in each of these models. As shown in S4 Table, both the p42 and p48 isoforms fit the additive model best (highest *R*^2^), whereas the recessive model outperformed in the p46 isoform regression (*R*^*2*^_*rec*_ = 0.57 vs. *R*^*2*^_*add*_ = 0.54). However, the difference of the *R*^*2*^ between the dominant and additive models in the p46 eQTL analysis is subtle. Also, the outperformance of the recessive model cannot be confirmed by qPCR (where the additive model has the highest *R*^*2*^). Therefore, we only reported the additive results in the main text. However, the alternative genetic models for the eQTL effect observed in different isoforms may reflect distinct disease mechanisms rendered by these isoforms, and thus detailed contribution of different isoforms on disease susceptibility warrant further functional study.

### Co-localization analysis

The co-localization analysis between genetic association and *cis*-eQTL results in the *OAS1* region was performed using eCAVIAR [53]. We used the *z*-scores (calculated by β/standard error) and the LD matrix (calculated using PLINK—r) from both the genetic association and *cis*-eQTL results as input and assumed one causal variant to obtain colocalization posterior probability (CLPP) scores for all the tested 453 variants in the *OAS1* region.

### RNA-seq experiments and analyses

Peripheral blood mRNA transcripts from 27 anti-Ro/SSA positive SS cases, 33 anti-Ro/SSA negative SS cases, and 30 healthy controls were isolated and measured as described above. RNA-seq was performed using the Illumina HiSeq 2000 employing standard procedures. Multiplexing of 6 samples per lane was utilized. Post sequence data were processed with Illumina Pipeline software v.1.7. Quality of raw sequence data was assessed using FASTQC. We assessed the quality of each sample using AQM [92] as described above. A total of 6 samples were removed from analysis due to significantly different expression patterns revealed by PC analysis. Raw FASTQ files were aligned to the human reference genome (hg19) using TopHat [57] that aligns the reads across splicing junctions independent of gene annotations, which benefits *de novo* detection of alternative splicing events. The total gene transcript level was determined by normalized read counts (raw read counts divided by estimated size factor) in DESeq [109].

To determine alternative splicing events, the reference-independent construction of the transcripts was performed using Cufflinks [58] to identify transcripts >1% of the most abundant isoform in each sample. We only kept the transcripts that were detected in more than 10% of the samples for further analysis. The previously annotated isoforms (p46, p42 and p48) and an un-annotated isoform identified across multiple samples (p44) were used as reference to reconstruct the isoforms of *OAS1*. The novel identified isoforms of *OAS1* were also checked manually in the Integrative Genomics Viewer (IGV) [110] to confirm the transcripts and cross-exon reads. The FPKM values calculated by Cufflinks were used to determine the expression levels of each isoform of *OAS1*.

### *OAS1* cloning and transfection

Total RNA was extracted using TRIzol reagents (Life Technologies) from EBV-immortalized B cells pre-selected for the presence of target *OAS1* isoforms based on the RNA-seq results from whole blood. Following DNase treatment (Life Technologies) and cDNA synthesis (iScript kit from Bio-Rad), full-length and truncated *OAS1* transcripts were amplified from cDNA using primer sets specific for the different *OAS1* isoforms and truncated forms (S3 Table). Each *OAS1* isoform transcript was individually cloned into pcDNA3.1 (Invitrogen) with an Xpress epitope tag at the 5'-terminus to facilitate the detection of transfected protein using Western-blot with anti-Xpress antibody. The plasmid was transfected into the HEK 293T cells using FuGENE transfection reagents (Promega) following manufacturer’s protocols.

### OAS1 protein levels determination by Western blot

The protein expression of OAS1 isoforms was evaluated in EBV-immortalised B cells from SS patients, four independent samples from each genotype group GG, GA and AA, treated or not treated with type I interferon (universal type I IFN, 1500 U/mL, for 24 hours). The cells were lysed in RIPA buffer and cell lysate protein concentration determined using the Qubit Protein Assay kit (Thermo Fisher Scientific). A total of 30 μg protein from each cell extract was separated on a 10% Bis-Tris gel (10% Criterion^™^ XT Bis-Tris Gel, BioRad, Cat #: 3450112) following the manufacturer’s instructions, the gels cut according to the weight of the OAS1 protein, and simultaneously transferred to a single PVDF membrane, thus ensuring the comparability of Western blot bands from all gels. The OAS1 isoforms were visualized using an anti-OAS1 antibody targeting the shared epitope (Rabbit polyclonal anti-human OAS1, Abcam, Cat #: ab86343) and ECL Prime Western Blotting Detection Reagents (Amersham, Cat #: RPN2232).

## Supporting information

S1 Fig Test of anti-Ro/SSA-specific genetic effect of rs10774671 with SS. (A) We stratified our SS case samples based on anti-Ro/SSA status and merged the samples from Dataset1 and Dataset2 to boost statistical power. We performed a *Z*-test based on regression coefficients where β1 and β2 correspond to the regression coefficients from anti-Ro/SSA positive and negative datasets, respectively, and SEβ1 and SEβ2 are standard errors of the regression coefficients. There is no significant difference of the genetic effects from rs10774671 on SS susceptibility between results from the anti-Ro/SSA positive dataset and anti-Ro/SSA negative dataset (*p* = 0.60). (B) Down-sampling tests for association of rs10774671 with SS in the anti-Ro/SSA positive dataset. The bar plot shows the distribution of *p* values from 10,000 permutation tests when down-sampling the cases in the anti-Ro/SSA positive dataset to n = 280 to match the case size in the anti-Ro/SSA negative dataset. The red line shows the *p* value of the association of rs10774671 with SS in the anti-Ro/SSA negative dataset (*P*_*neg*_ = 1.28×10^−2^). We calculated an empirical *p* value to compare *P*_*neg*_ with the down-sampled *p* values based on the rank of the *P*_*neg*_ within the 10,000 permutation *p* values, and found *P*_*neg*_ is not significantly deviated from the permutation tests from the anti-Ro/SSA positive dataset (*P*_*emp*_ = 0.22).
(TIF)

S2 Fig Replication of RNA-seq results for the transcript levels of *OAS1* isoforms using quantitative real-time PCR. The transcript levels of each *OAS1* isoform were determined by real-time PCR using primer sets targeting the specific *OAS1* isoform (S3 Table). The transcript levels of *OAS1* isoforms were normalized to the housekeeping gene *HMBS*. Consistent with the RNA-seq results, the SS-associated risk allele A of rs10774671 was correlated with increased expression of the (A) p42, (B) p48, and (C) p44 isoforms but decreased levels of (D) p46 (*P* values were determined using Kruskal-Wallis test not assuming equal standard deviation across different groups). The error bar indicates standard error of the mean. There is no error bar in the AA group from p48 or GG group from p44, as the transcripts were not detectable using real-time PCR in these samples. (GG: n = 6; GA: n = 7; AA: n = 9)
(TIF)

S3 Fig Test of anti-Ro/SSA-specific eQTL effect of rs10774671. (A) we performed eQTL analyses using the RNA-seq data in anti-Ro/SSA positive patients (n = 27) and anti-Ro/SSA negative patients (n = 30) separately. We performed linear regression on the expression of the four isoforms of *OAS1* while adjusting for sex in the two subsets of samples. We performed a *Z*-test to determine whether the eQTL effects are different between anti-Ro/SSA positive group and anti-Ro/SSA negative group. We did not find any significant difference of the eQTL effects between the two sub-groups. (B) we performed a linear regression analysis in all SS patients (n = 57) using an interaction term (genotype * anti-Ro) as an independent variable while adjusting for sex (expression ~ genotype * anti-Ro + sex). In the analyses for the p46, p42, and p48 isoforms, both the autoantibody status and the genotype are significantly associated with *OAS1* isoform expression. The *OAS1* transcripts are part of the interferon signature, which is well-documented to be correlated with auto-Ro/SSA status; however, the variation of *OAS1* isoform expression is explained more by the genotype of rs10774671 compared to anti-Ro/SSA status. Also, none of the linear regression models is significant for the (genotype * anti-Ro) term, which indicates that the anti-Ro/SSA positivity does not influence the genetic effect of rs10774671 on *OAS1* isoform expression.
(TIF)

S4 Fig Total transcript levels of *OAS1* in RNA-seq from subjects with different genotypes of rs10774671. The total *OAS1* transcript levels regardless of the isoforms was determined using the normalized read counts mapped to the *OAS1* region from RNA-seq. (A) Statistically significant differences were observed for the total *OAS1* transcript expression between the case and control groups carrying the same genotype (GA and AA group). (B) The overexpression of total *OAS1* in SS cases was explained by subjects who are anti-Ro/SSA positive, as significant higher expression of total *OAS1* was observed in anti-Ro/SSA positive cases compared to both controls and anti-Ro/SSA negative cases in the GA and AA groups. *P* values were determined using two-tailed *t* test (Significance level: * *P*<0.05; ** *P*<0.01; *** *P*<0.001; **** *P*<0.0001). The Mean±SEM of each group are plotted in red.
(TIF)

S5 Fig Influence of the 3'-terminus of each *OAS1* isoform on protein expression. (A) The 3' alternatively spliced terminus of different *OAS1* isoforms (indicated by Δ) were linked to the 3'-end of GFP followed by transfection into HEK 293T cells. GFP was cloned into pcDNA3.1 first and then ligated with *OAS1* 3' end cutting from the pBluescript II KS plasmid carrying individual *OAS1* isoform clones (S3 Table). (B) Normal expression of GFP was observed when linked with the 3'-terminus from the normally spliced isoform p46 (GFP-Δp46); while the 3'-terminus from the p48 and p44 isoforms resulted in decreased protein expression of GFP. (C) Zoomed in photos show that while GFP-Δp46 was universally expressed, 3'-end of the three alternatively spliced isoforms (p42, p48, and p44) due to the SS-associated risk allele A of rs10774671 seem to alter GFP distribution into certain organelles of the cell (arrows). Further detailed study is needed to investigate which specific organelle does each alternatively spliced isoform is enriched in.
(TIF)

S6 Fig Associations of rs10774671 with *OAS1* expression from the GTEx database. (A) We found significant eQTLs of rs10774671 on the expression of *OAS1* in 5 tissues from the GTEx database. (B,C) The eQTL effects of rs10774671 on the *OAS1* expression in whole blood and esophagus mucosa. The right part of the figure shows isoform expression of the p46 and p42 isoforms in whole blood and esophagus mucosa.
(TIF)

S7 Fig Comparison of blood cell type composition between SS patients and healthy controls. In order to determine whether the overexpression of IFN signature genes in SS patients was due to elevated numbers of immune cells in whole blood, we compared the results from the differential cell counts derived from complete blood between SS patients and healthy controls used in our gene expression study. Lymphocyte counts were the only immune cell type with cell counts significantly different between SS patients and controls. As expected, the lymphocyte counts were lower in SS patients compared to controls; thus, they do not explain the overexpression of IFN-inducible genes in patients. Lymphopenia has been observed in multiple autoimmune diseases, including SS [111], likely through the mechanism of “two-hit model of autoimmunity”, where both lymphopenia and the aberrant responsiveness of T cells to *TGF-β* signaling are required to trigger the development of autoimmune disease [112, 113]. It is unclear whether or how type I IFN signaling may be involved in this process as a link between innate and adaptive immune responses. We propose that the IFN signatures are more related to the mechanisms that involve viral infection and autoantibody production, rather than influencing lymphocyte tolerance and homeostasis as proposed in the “two-hit model of autoimmunity”. However, both mechanisms may interact with each other during the development of autoimmune diseases.
(TIF)

S8 Fig Pipeline and results of the QC and normalization procedures for the transcriptome profiling study. (A) A stringent QC process was applied to the microarray data prior to analyses. Quality assessments for both samples and probes were performed through a series of processes using packages in the R suite. Normalization to the whole dataset was performed as a routine procedure for whole transcriptome analysis. The differentially expressed genes between SS cases and controls were identified using a significant cuff-off of *q*<0.05 and absolute FC > 2 before the downstream clustering and *cis*-eQTL analyses. (B) The expression levels of all probes (y-axis) in each individual (x-axis) were plotted for raw data, after QC and normalization, and after removing the batch effect using the ComBat program.
(TIF)

S9 Fig Assessments of population stratification using principal component analysis. The population ancestry was determined for each sample prior to analysis to keep only the samples of European ancestry. Plots of PC2 vs. PC1 from the principal component analyses for (A) the initial genetic association study (Dataset 1) and (B) replication study (Dataset 2) are shown. The analyses were performed along with samples from the HapMap cohort. The final samples used in the respective analyses are shown as black dots.
(TIF)

S1 Table Results of transcriptome profiling, genetic association, and cis-eQTL analyses for the dysregulated genes in SS. (XLSX)

S2 Table Results of co-localization analysis using eCAVIAR. (XLSX)

S3 Table Sequence of the primers used in this study. (XLSX)

S4 Table Results of eQTL analyses for *OAS1* isoforms using different genetic models. (XLSX)

S5 Table Microarray data. Microarray data that passed QC.
(TXT)

S6 Table Microarray sample info. metadata information for samples in the microarray. (TXT)

S7 Table RNAseq data. Normalized OAS1 isoform expression.
(TXT)

S8 Table Genotype OAS1. Genotypes of all the variants in the OAS1 region in the eQTL analysis.
(TXT)

S1 Text Membership of UK Primary Sjögren's Syndrome Registry. (PDF)
