# Supplementary figures and images for: Identification of a Sjögren's syndrome susceptibility locus at OAS1 that influences isoform switching, protein expression, and responsiveness to type I interferons
Source: PLoS Genet. 2017 Jun 22;13(6):e1006820. doi: 10.1371/journal.pgen.1006820 (PMC5501660; doi:10.1371/journal.pgen.1006820)

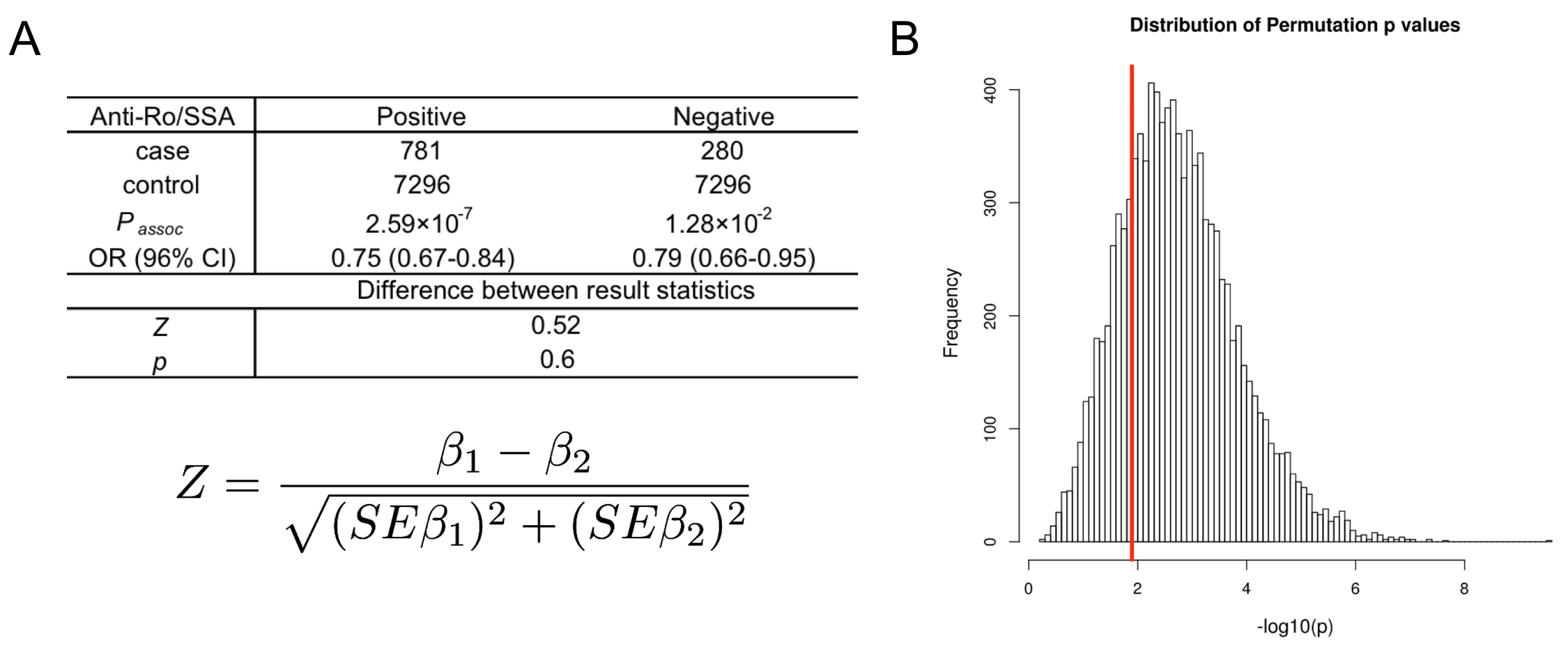

Supplement: S1 Fig — (A) We stratified our SS case samples based on anti-Ro/SSA status and merged the samples from Dataset1 and Dataset2 to boost statistical power. We performed a Z-test based on regression coefficients where β1 and β2 correspond to the regression coefficients from anti-Ro/SSA positive and negative datasets, respectively, and SEβ1 and SEβ2 are standard errors of the regression coefficients. There is no significant difference of the genetic effects from rs10774671 on SS susceptibility between results from the anti-Ro/SSA positive dataset and anti-Ro/SSA negative dataset (p = 0.60). (B) Down-sampling tests for association of rs10774671 with SS in the anti-Ro/SSA positive dataset. The bar plot shows the distribution of p values from 10,000 permutation tests when down-sampling the cases in the anti-Ro/SSA positive dataset to n = 280 to match the case size in the anti-Ro/SSA negative dataset. The red line shows the p value of the association of rs10774671 with SS in the anti-Ro/SSA negative dataset (Pneg = 1.28×10^−2^). We calculated an empirical p value to compare Pneg with the down-sampled p values based on the rank of the Pneg within the 10,000 permutation p values, and found Pneg is not significantly deviated from the permutation tests from the anti-Ro/SSA positive dataset (Pemp = 0.22). (TIF) [file pgen.1006820.s001.tif]

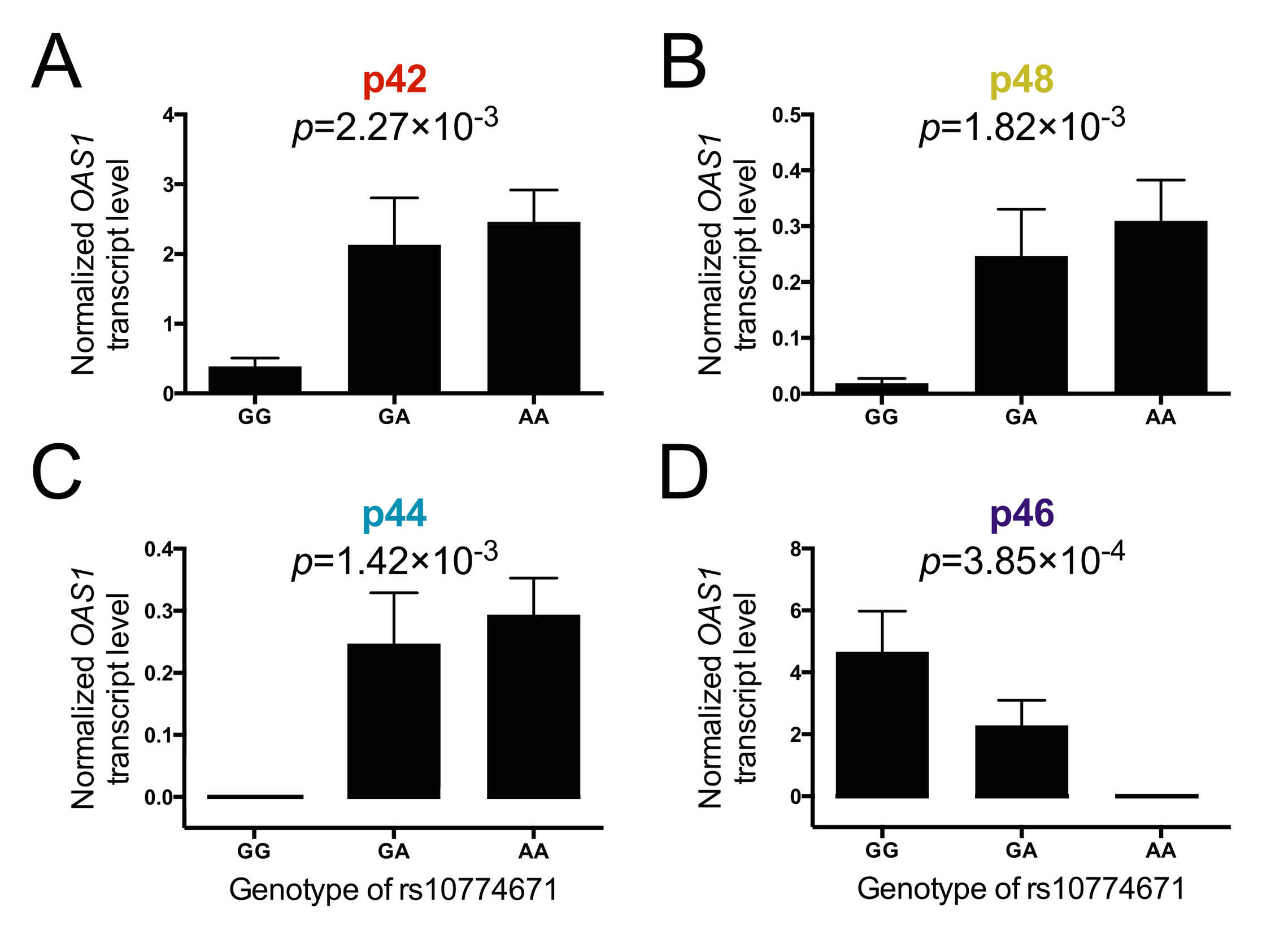

Supplement: S2 Fig — The transcript levels of each OAS1 isoform were determined by real-time PCR using primer sets targeting the specific OAS1 isoform (S3 Table). The transcript levels of OAS1 isoforms were normalized to the housekeeping gene HMBS. Consistent with the RNA-seq results, the SS-associated risk allele A of rs10774671 was correlated with increased expression of the (A) p42, (B) p48, and (C) p44 isoforms but decreased levels of (D) p46 (P values were determined using Kruskal-Wallis test not assuming equal standard deviation across different groups). The error bar indicates standard error of the mean. There is no error bar in the AA group from p48 or GG group from p44, as the transcripts were not detectable using real-time PCR in these samples. (GG: n = 6; GA: n = 7; AA: n = 9) (TIF) [file pgen.1006820.s002.tif]

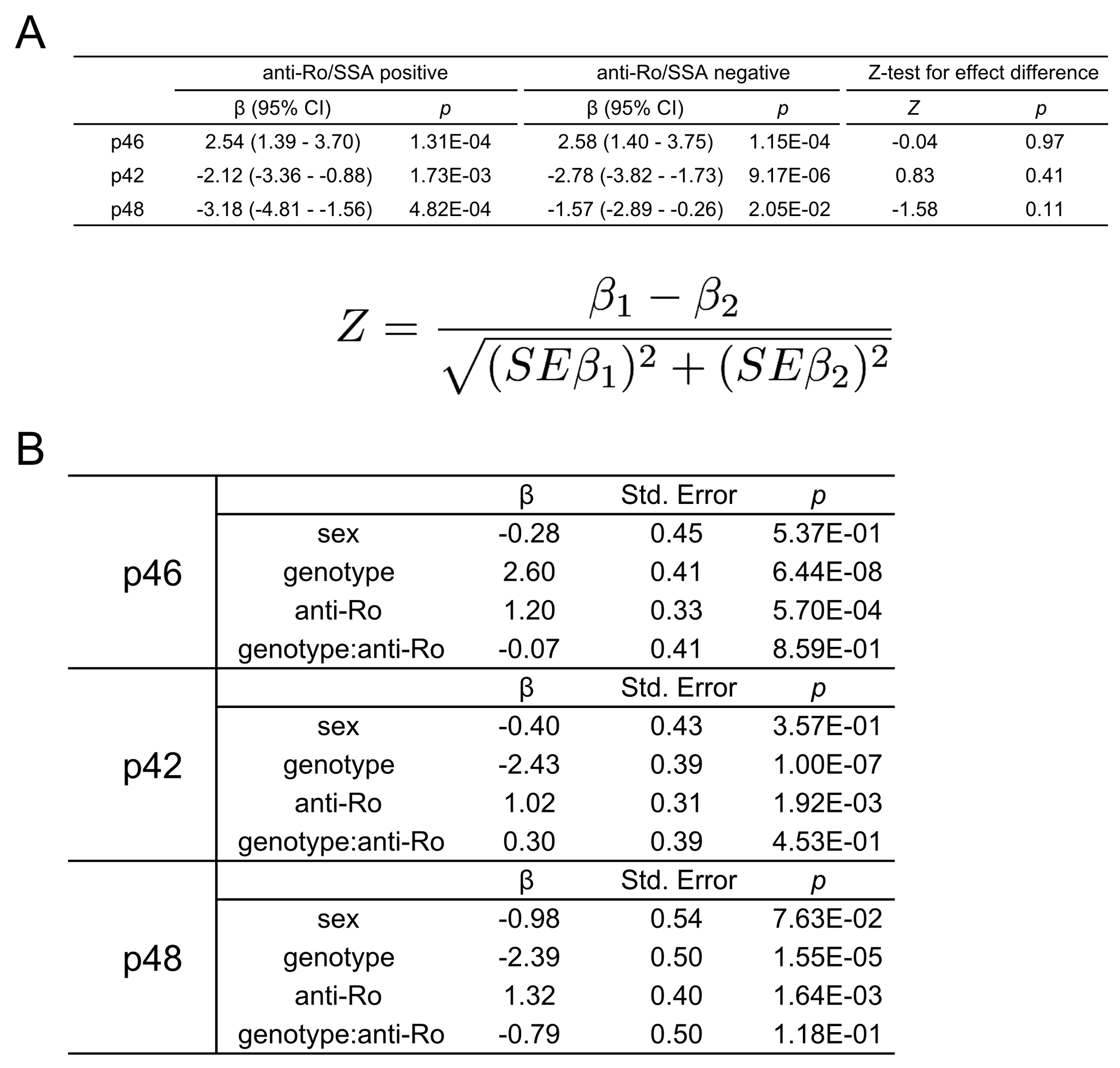

Supplement: S3 Fig — (A) we performed eQTL analyses using the RNA-seq data in anti-Ro/SSA positive patients (n = 27) and anti-Ro/SSA negative patients (n = 30) separately. We performed linear regression on the expression of the four isoforms of OAS1 while adjusting for sex in the two subsets of samples. We performed a Z-test to determine whether the eQTL effects are different between anti-Ro/SSA positive group and anti-Ro/SSA negative group. We did not find any significant difference of the eQTL effects between the two sub-groups. (B) we performed a linear regression analysis in all SS patients (n = 57) using an interaction term (genotype * anti-Ro) as an independent variable while adjusting for sex (expression ~ genotype * anti-Ro + sex). In the analyses for the p46, p42, and p48 isoforms, both the autoantibody status and the genotype are significantly associated with OAS1 isoform expression. The OAS1 transcripts are part of the interferon signature, which is well-documented to be correlated with auto-Ro/SSA status; however, the variation of OAS1 isoform expression is explained more by the genotype of rs10774671 compared to anti-Ro/SSA status. Also, none of the linear regression models is significant for the (genotype * anti-Ro) term, which indicates that the anti-Ro/SSA positivity does not influence the genetic effect of rs10774671 on OAS1 isoform expression. (TIF) [file pgen.1006820.s003.tif]

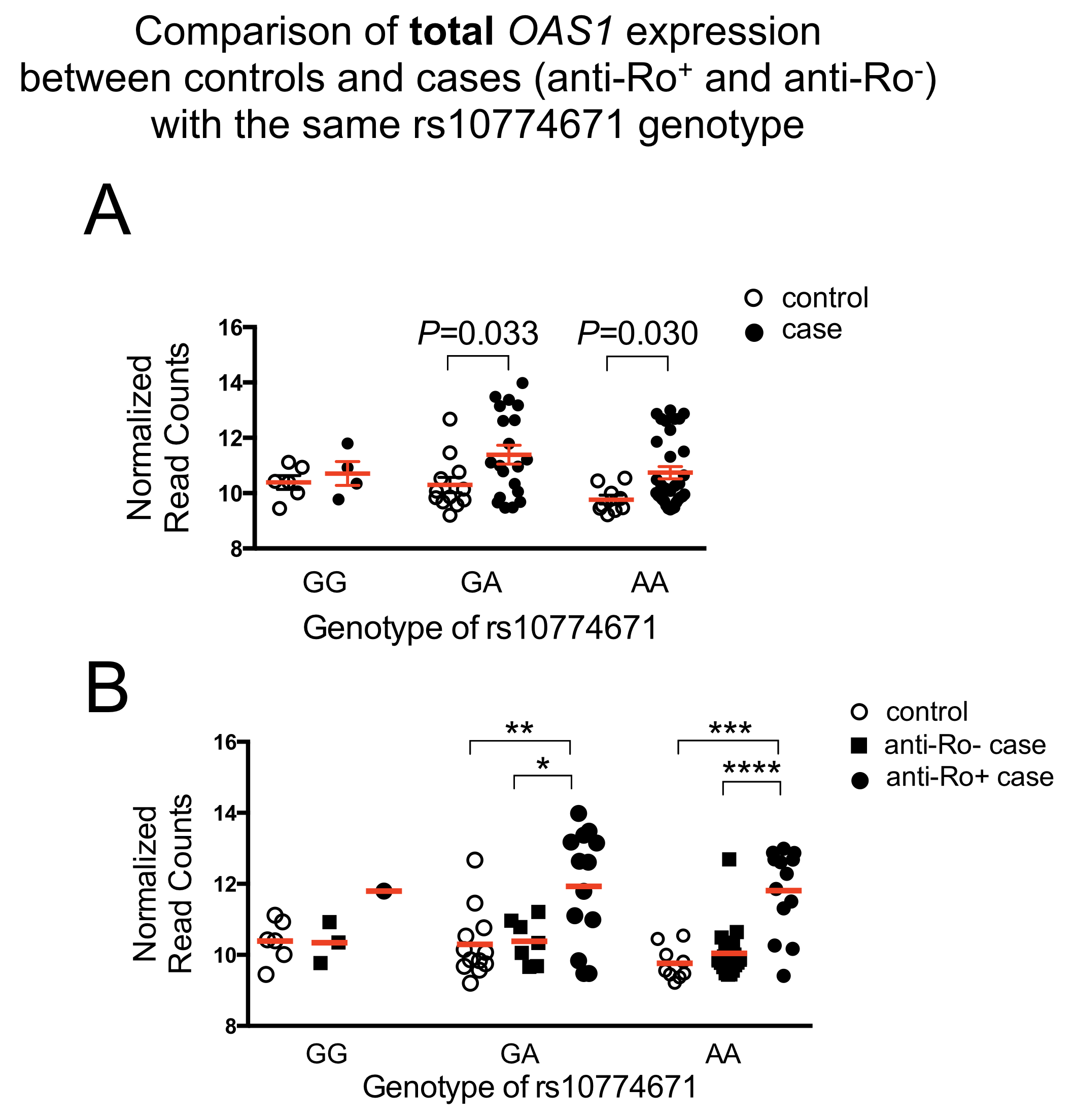

Supplement: S4 Fig — The total OAS1 transcript levels regardless of the isoforms was determined using the normalized read counts mapped to the OAS1 region from RNA-seq. (A) Statistically significant differences were observed for the total OAS1 transcript expression between the case and control groups carrying the same genotype (GA and AA group). (B) The overexpression of total OAS1 in SS cases was explained by subjects who are anti-Ro/SSA positive, as significant higher expression of total OAS1 was observed in anti-Ro/SSA positive cases compared to both controls and anti-Ro/SSA negative cases in the GA and AA groups. P values were determined using two-tailed t test (Significance level: * P<0.05; ** P<0.01; *** P<0.001; **** P<0.0001). The Mean±SEM of each group are plotted in red. (TIF) [file pgen.1006820.s004.tif]

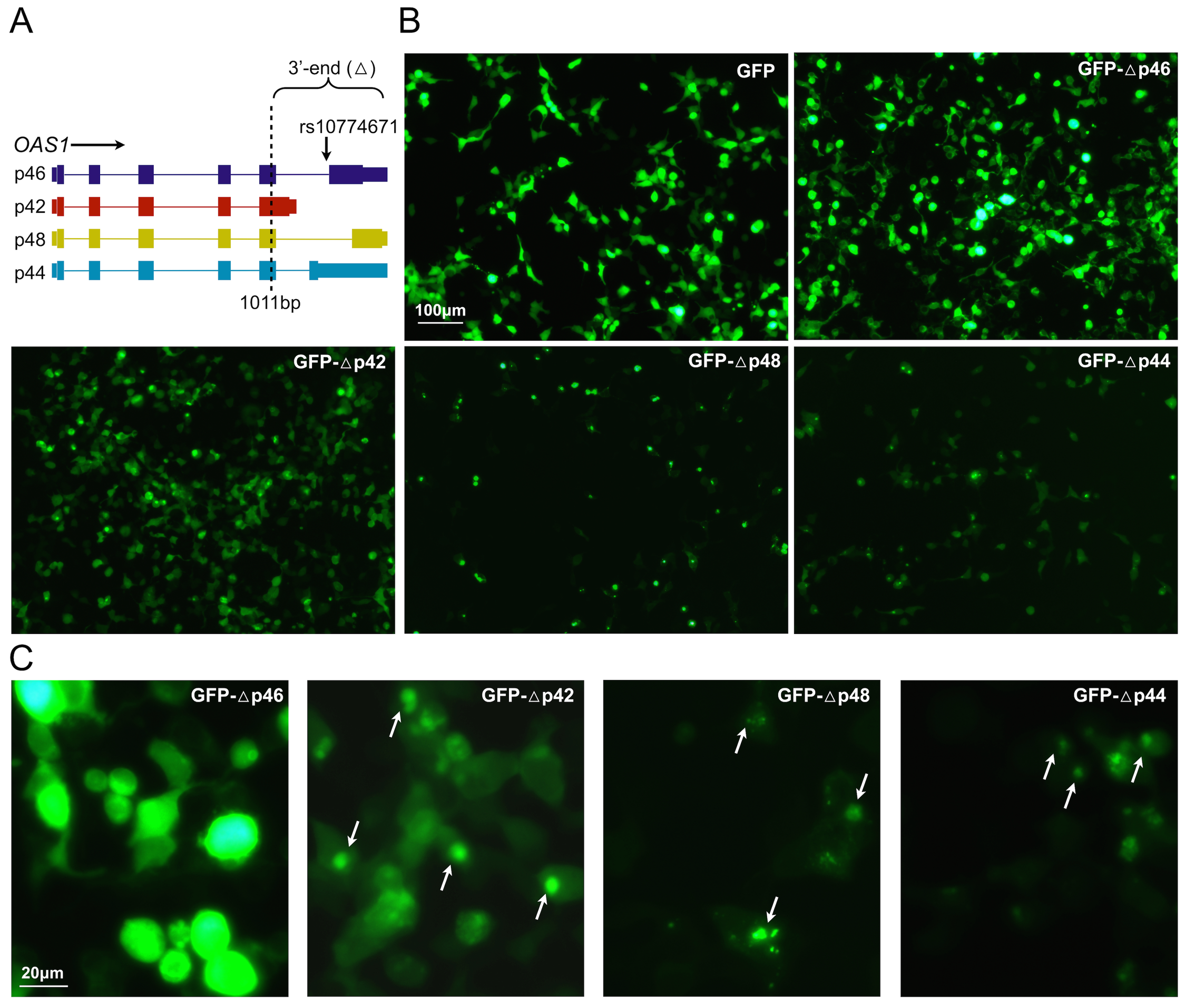

Supplement: S5 Fig — (A) The 3' alternatively spliced terminus of different OAS1 isoforms (indicated by Δ) were linked to the 3'-end of GFP followed by transfection into HEK 293T cells. GFP was cloned into pcDNA3.1 first and then ligated with OAS1 3' end cutting from the pBluescript II KS plasmid carrying individual OAS1 isoform clones (S3 Table). (B) Normal expression of GFP was observed when linked with the 3'-terminus from the normally spliced isoform p46 (GFP-Δp46); while the 3'-terminus from the p48 and p44 isoforms resulted in decreased protein expression of GFP. (C) Zoomed in photos show that while GFP-Δp46 was universally expressed, 3'-end of the three alternatively spliced isoforms (p42, p48, and p44) due to the SS-associated risk allele A of rs10774671 seem to alter GFP distribution into certain organelles of the cell (arrows). Further detailed study is needed to investigate which specific organelle does each alternatively spliced isoform is enriched in. (TIF) [file pgen.1006820.s005.tif]

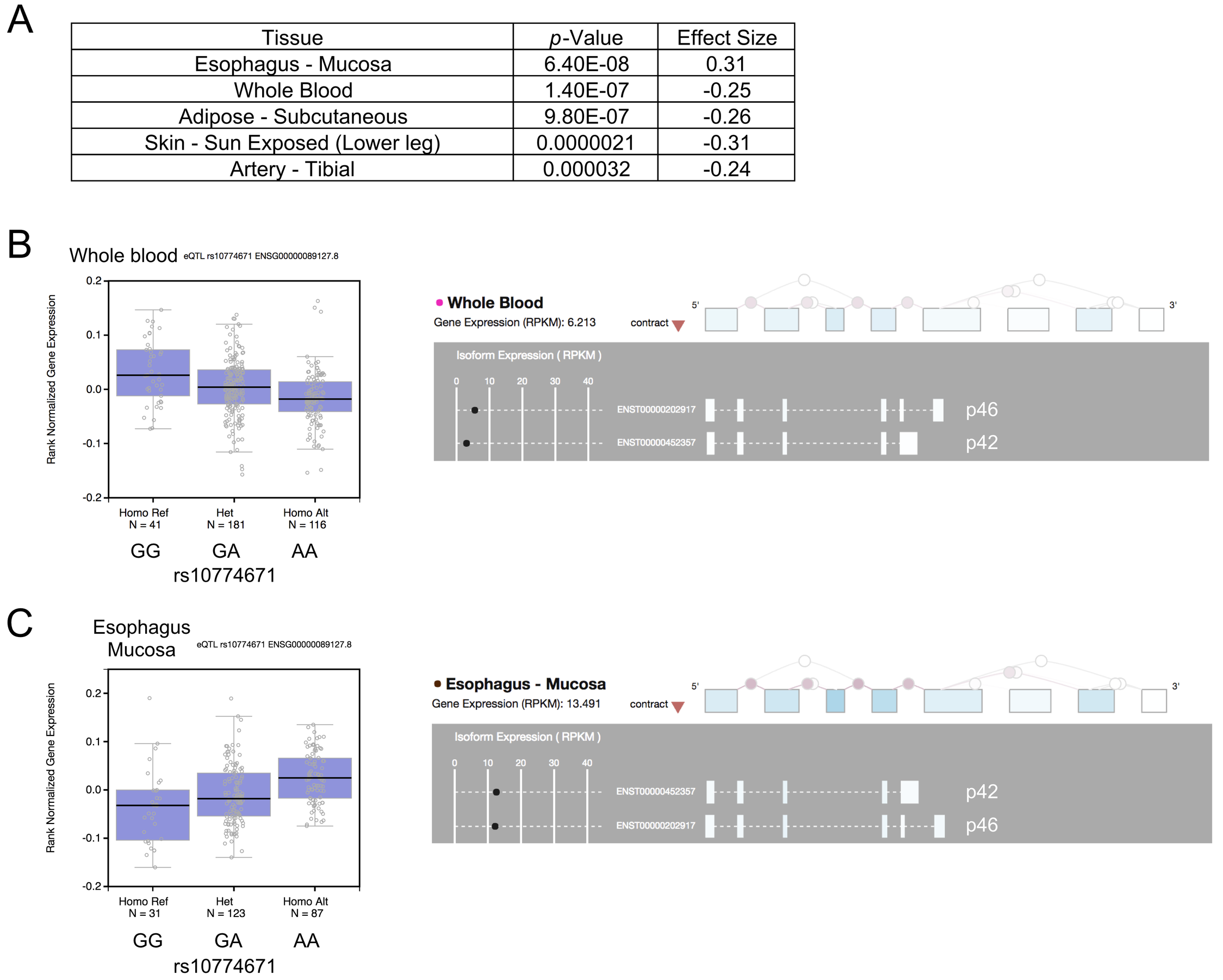

Supplement: S6 Fig — (A) We found significant eQTLs of rs10774671 on the expression of OAS1 in 5 tissues from the GTEx database. (B,C) The eQTL effects of rs10774671 on the OAS1 expression in whole blood and esophagus mucosa. The right part of the figure shows isoform expression of the p46 and p42 isoforms in whole blood and esophagus mucosa. (TIF) [file pgen.1006820.s006.tif]

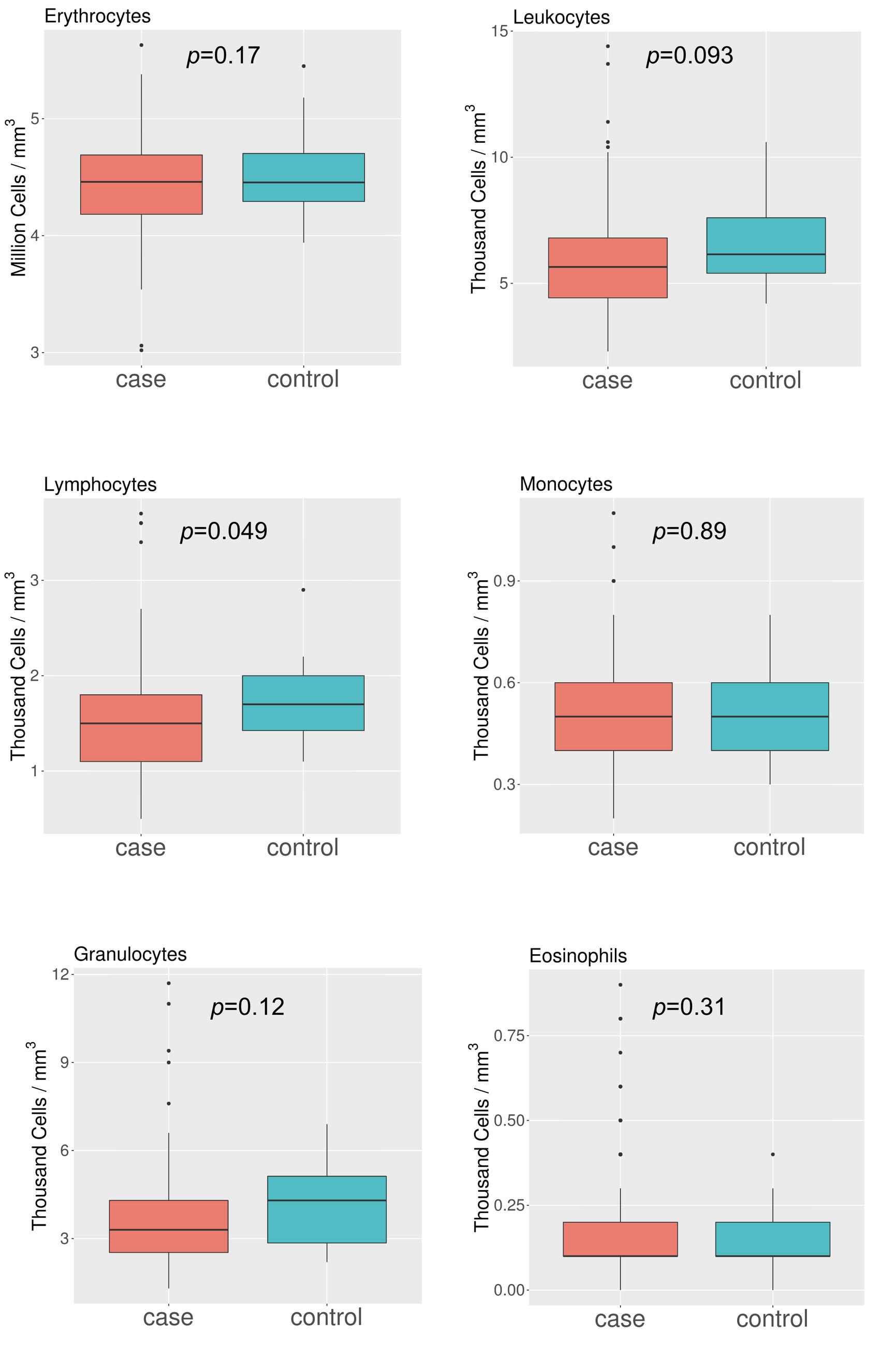

Supplement: S7 Fig — In order to determine whether the overexpression of IFN signature genes in SS patients was due to elevated numbers of immune cells in whole blood, we compared the results from the differential cell counts derived from complete blood between SS patients and healthy controls used in our gene expression study. Lymphocyte counts were the only immune cell type with cell counts significantly different between SS patients and controls. As expected, the lymphocyte counts were lower in SS patients compared to controls; thus, they do not explain the overexpression of IFN-inducible genes in patients. Lymphopenia has been observed in multiple autoimmune diseases, including SS [111], likely through the mechanism of “two-hit model of autoimmunity”, where both lymphopenia and the aberrant responsiveness of T cells to TGF-β signaling are required to trigger the development of autoimmune disease [112, 113]. It is unclear whether or how type I IFN signaling may be involved in this process as a link between innate and adaptive immune responses. We propose that the IFN signatures are more related to the mechanisms that involve viral infection and autoantibody production, rather than influencing lymphocyte tolerance and homeostasis as proposed in the “two-hit model of autoimmunity”. However, both mechanisms may interact with each other during the development of autoimmune diseases. (TIF) [file pgen.1006820.s007.tif]

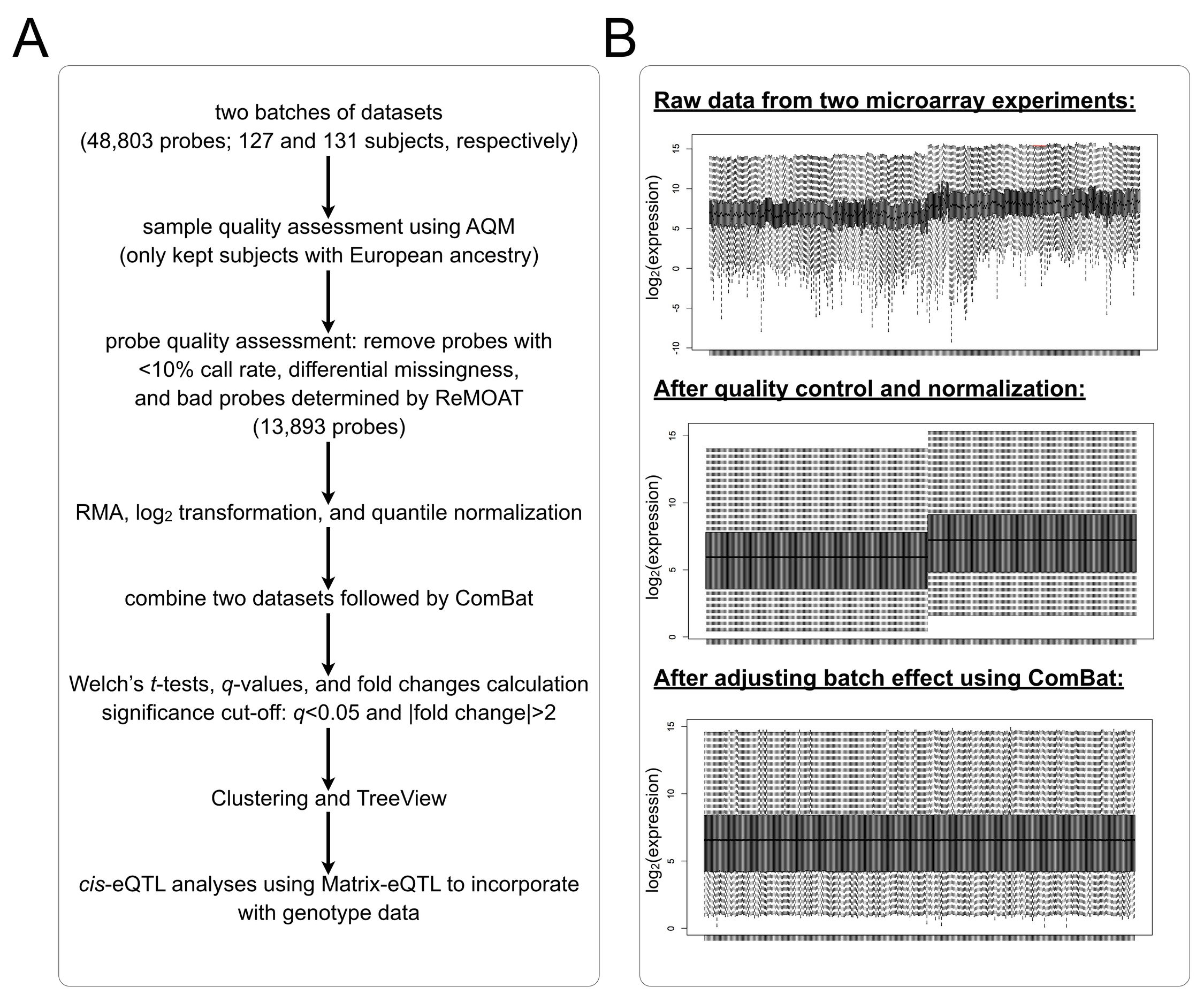

Supplement: S8 Fig — (A) A stringent QC process was applied to the microarray data prior to analyses. Quality assessments for both samples and probes were performed through a series of processes using packages in the R suite. Normalization to the whole dataset was performed as a routine procedure for whole transcriptome analysis. The differentially expressed genes between SS cases and controls were identified using a significant cuff-off of q<0.05 and absolute FC > 2 before the downstream clustering and cis-eQTL analyses. (B) The expression levels of all probes (y-axis) in each individual (x-axis) were plotted for raw data, after QC and normalization, and after removing the batch effect using the ComBat program. (TIF) [file pgen.1006820.s008.tif]

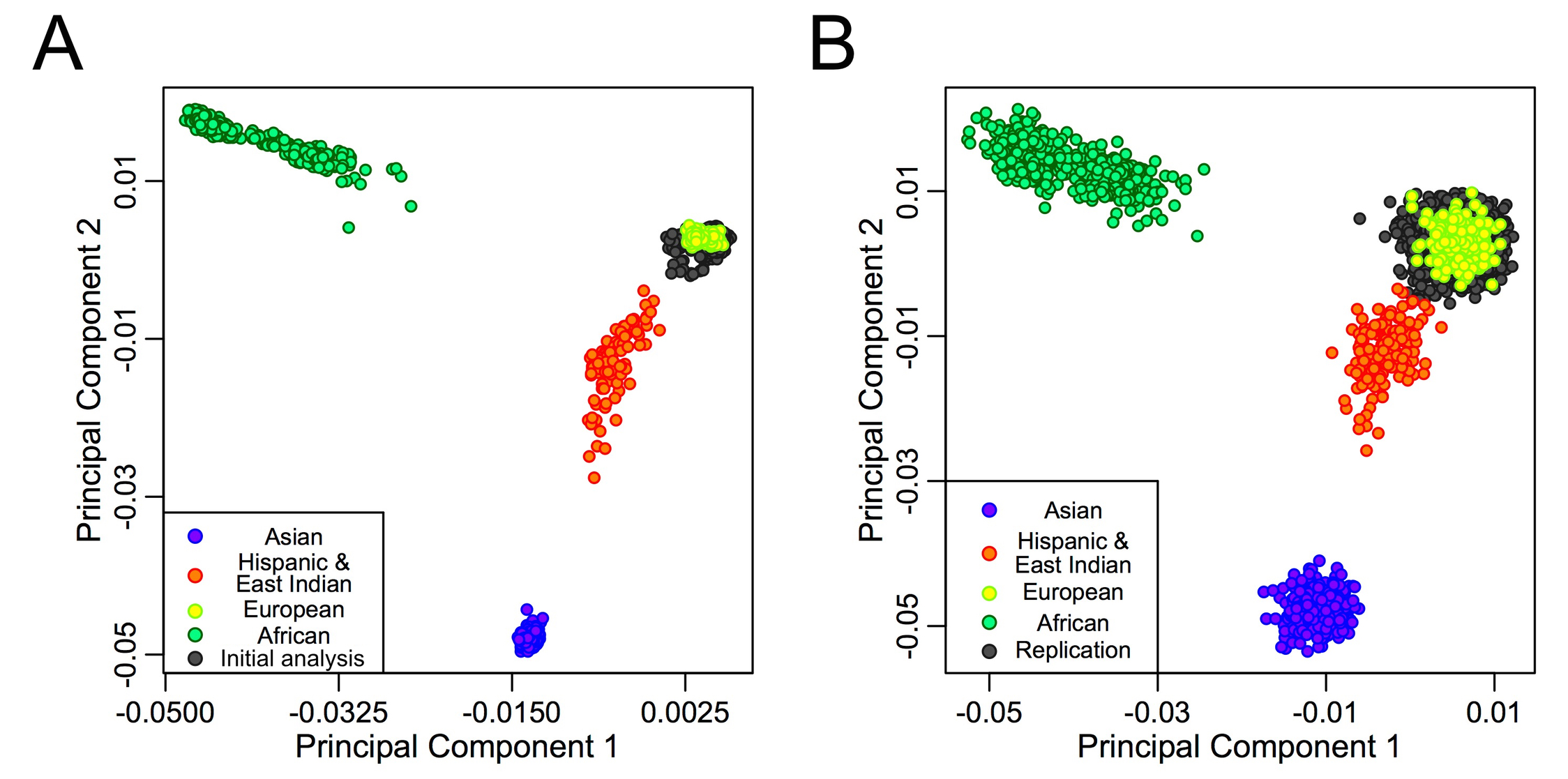

Supplement: S9 Fig — The population ancestry was determined for each sample prior to analysis to keep only the samples of European ancestry. Plots of PC2 vs. PC1 from the principal component analyses for (A) the initial genetic association study (Dataset 1) and (B) replication study (Dataset 2) are shown. The analyses were performed along with samples from the HapMap cohort. The final samples used in the respective analyses are shown as black dots. (TIF) [file pgen.1006820.s009.tif]
