# Supplementary material for: Identification of a Sjögren's syndrome susceptibility locus at OAS1 that influences isoform switching, protein expression, and responsiveness to type I interferons
Source: PLoS Genet. 2017 Jun 22;13(6):e1006820. doi: 10.1371/journal.pgen.1006820 (PMC5501660; doi:10.1371/journal.pgen.1006820)
Supplement: S1 Text — (PDF) [file pgen.1006820.s018.pdf]

The UK Primary Sjogren's Syndrome Registry (UKPSSR) is composed of the following individuals (in alphabetical order of their affiliations):

Elalaine C Bacabac, Helen Frankland, Robert Moots (Aintree University Hospitals); Kuntal Chadravarty, Shamin Lamabadusuriya (Barking, Havering and Redbridge NHS Trust); Michele Bombardieri, Constantino Pitzalis, Nurhan Sutcliffe, Celia Breston (Bart and the London NHS Trust); Nagui Gendi, Karen Culfear (Basildon Hospital); Claire Riddell (Belfast Health and Social Care Trust); John Hamburger, Andrea Richards (Birmingham Dental Hospital); Saaeha Rauz (Birmingham & Midland Eye Centre); Sue Brailsford, Joanne Dasgin (Birmingham University Hospital); Joanne Logan, Diarmuid Mulherin (Cannock Chase Hospital); Jacqueline Andrews, Paul Emery, Alison McManus, Colin Pease, David Pickles (Chapel Allerton Hospital, Leeds); Alison Booth, Marian Regan (Derbyshire Royal Infirmary); Jon King, Amanda Holt (Derriford Hospital, Plymouth); Theodoros Dimitroulas, Lucy Kadiki, Daljit Kaur, George Kitas (Dudley Group of Hospitals NHS Foundation Trust); Abdul Khan, Tracey Cosier (East Kent Hospitals University); Panthakalam, Kelly Mintrim (East Sussex Healthcare NHS); Mark Lloyd, Lisa Moore (Frimley Park Hospital); Esther Gordon, Cathy Lawson (Harrogate District Foundation Trust Hospital); Monica Gupta, John Hunter, Lesley Stirton (Gartnavel General Hospital, Glasgow); Gill Ortiz, Elizabeth Price; Suzannah Pelger (Great Western Hospital); Claire Gorman, Balinder Hans (Homerton Hospital); Gavin Clunie, Suzanne Lane, Ginny Rose, Sue Cuckow (Ipswich Hospital NHS Trust); Michael Batley, Ruby Einosas (Maidstone Hospital); Susan Knight, Deborah Symmons, Beverley Jones (Macclesfield District General Hospital & Arthritis Research UK Epidemiology Unit, Manchester); Andrew Carr, Suzanne Edgar, Francisco Figuereido, Heather Foggo, Dennis Lendrem, Iain Macleod, Sheryl Mitchell, Wan-Fai Ng, Christine Downie, Jessica Tarn, James Locke, Shereen Al-Ali, Sarah Legg, Kamran Mirza (Newcastle upon Tyne Hospitals NHS Foundation Trust and Newcastle University); Adrian Jones, Peter Lanyon, Alice Muir (Nottingham University Hospital); Paula White, Steven Young-Min (Portsmouth Hospitals NHS Trust); Susan Pugmire, Saravanan Vadivelu (Queen's Elizabeth Hospital, Gateshead); Annie Cooper, Marianne Watkins (Royal Hampshire County Hospital); Anne Field, Stephen

Kaye, Devesh Mewar, Patricia Medcalf, Pamela Tomlinson, Debbie Whiteside (Royal Liverpool University Hospital); Neil McHugh, John Pauling, Julie James, Andrea Dowden (Royal National Hospital for Rheumatic Diseases); Mohammed Akil, Jayne McDermott, Olivia Godia (Royal Sheffield Hospital); David Coady, Elizabeth Kidd, Lynne Palmer (Royal Sunderland Hospital); Sarah Bartrum, Dee Mead (Salisbury District Hospital); Bhaskar Dasgupta, Victoria Katsande, Pamela Long, Olivia Godia (Southend University Hospital); Erin Vermaak, Janet Turner (Staffordshire Stoke-on-Trent Partnership); Usha Chandra, Kirsten MacKay (Torbay Hospital); Stefano Fedele, Ada Ferenkeh-Koroma, Ian Giles, David Isenberg, Helena Marconnell, Nyarko Ahwireng, Stephen Porter (University College Hospital & Eastman Dental Institute); Paul Allcoat, John McLaren (Whyteman's Brae Hospital, Kirkaldy).
